# Rescuing ACE2‐Deficiency‐Mediated Nucleus Pulposus Senescence and Intervertebral Disc Degeneration by a Nanotopology‐Enhanced RNAi System

**DOI:** 10.1002/advs.202412908

**Published:** 2025-01-13

**Authors:** Kaiqiang Sun, Zijian Kang, Chen Yan, Yan Yu, Linhui Han, Yangyang Shi, Liang Chen, Jiangang Shi, Yu Chen, Jingchuan Sun

**Affiliations:** ^1^ Department of Orthopedic Surgery Changzheng Hospital Naval Medical University Shanghai 200003 P. R. China; ^2^ Department of Orthopedics Naval Medical Center of PLA Shanghai 200052 P. R. China; ^3^ Department of Rheumatology and Immunology Shanghai Sixth People's Hospital Shanghai Jiao Tong University School of Medicine Shanghai 200233 P. R. China; ^4^ State Key Laboratory of Molecular Engineering of Polymers Fudan University Shanghai 200433 P.R. China; ^5^ Materdicine Lab School of Life Sciences Shanghai University Shanghai 200444 P. R. China

**Keywords:** Intervertebral disc degeneration, Cellular senescnece, Nanotopology‐enhanced RNAi system, ACE2, Gene therapy

## Abstract

Nucleus pulposus cell (NPC) senescence contributes to intervertebral disc degeneration (IVDD). However, the underlying molecular mechanisms are not fully understood. In this study, it is demonstrated that angiotensin‐converting enzyme 2 (ACE2) counteracted the aging of NPCs and IVDD at the cellular and physiological levels. The expression of ACE2 correlates negatively with the degree of NPC senescence and IVDD. Using both loss‐ and gain‐of‐function mouse models, it is revealed that ACE2 deficiency increased the senescence of NPCs and exacerbated injury‐ or instability‐induced IVDD, whereas ACE2 overexpression counteracted these detrimental effects. Mechanistically, integrated analysis of single‐cell and bulk transcriptomics shows that ACE2 deficiency results in the activation of TGFβ2/Smads signaling pathway and the transcription of Serpine1, ultimately triggering NPC senescence and IVDD. A nanomedical delivery system (virus‐like nanovectors, VNs) composed of nanovectors with a virus‐like surface topology and small interfering RNA targeting Serpine1 (VN‐siSer) is developed. With nanotopology‐enhanced transfection efficiency, RNA‐interfering treatment by VN‐siSer effectively alleviated NPC senescence and IVDD at both the cellular and animal levels. Overall, the data reveal the underlying mechanisms of ACE2 in NPC senescence and IVDD pathogenesis and propose a distinct paradigm of precise nanomedical senescence‐blockade RNAi for IVDD treatment.

## Graphical abstract

ACE2 deficiency results in nucleus pulposus cell senescence via activating the TGFβ2/Smads signaling pathway and the transcription of Serpine1. A nanotopology‐enhanced RNAi system is developed for effectively alleviating nucleus pulposus cell senescence at both cellular and animal levels and rescuing intervertebral disc degeneration.

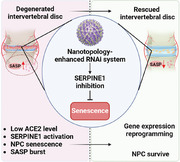

## 1 Introduction

Low back pain (LBP), which affects over 80% of adults during their lifetime and results in considerable disability and socioeconomic burden, is closely associated with intervertebral disc degeneration (IVDD).^[^ ^1^ ^]^ As one of the major spine degeneration‐related diseases, IVDD is characterized by the aging of nucleus pulposus cells (NPCs) and disequilibrium of the extracellular matrix (ECM).^[^ ^2^ ^]^ Two primary modalities are clinically utilized in the management of IVDD: conservative treatment and surgical intervention.^[^ ^3^ ^]^ Conservative treatment predominantly involves the administration of nonsteroidal anti‐inflammatory drugs (NSAIDs) and physical therapy, aiming to relieve pain in patients while negligibly impeding degeneration progression.^[^ ^4^ ^]^ Surgical treatments such as intervertebral disc removal, spinal fusion, and artificial disc replacement have demonstrated effectiveness in providing satisfactory pain relief and enhancing spinal stability. However, they are restricted by their high invasiveness and potential risks of adjacent disc degeneration. Therefore, there is an urgent need to explore suitable therapeutic modalities to resolve the underlying pathology of IVDD.

RNA‐interfering (RNAi) therapy offers new possibilities for treating refractory diseases by selectively manipulating the expression of any gene of interest.^[^ ^5^ ^]^ Nevertheless, the practical application of RNAi therapy in IVDD has been severely hampered by the lack of specific therapeutic targets and satisfactory delivery systems.^[^ ^6^ ^]^ On the one hand, the intricate pathological mechanisms underlying IVDD remain elusive to some extent, thus limiting the identification of molecular targets essential for precisely targeted RNAi therapy. However, effective RNA delivery into the cytoplasm requires a suitable vector to overcome the formidable extracellular and intracellular barriers. Conventional virus‐based vectors have inherent drawbacks, such as off‐target effects, virus‐induced immunogenicity, unwanted genomic integration, and mutational variability. With the development of nanotechnology and nanomedicine, researchers have fueled the search for alternative delivery vehicles for safer RNAi therapy.^[^ ^7^ ^]^ However, the endocytosis of non‐viral nanovectors is heavily influenced by their physicochemical properties, underscoring the importance of tailored design and fabrication of nanovectors to enhance transfection efficiency. Considering these two critical factors, it is imperative to identify effective therapeutic targets and develop specialized nanovectors for potent IVDD interventions.

Concerning new potential therapeutic targets, accumulating evidence highlights a heightened presence of senescent NPCs in IVDD.^[^ ^8^ ^]^ NPC senescence is an irreversible cell cycle arrest triggered by multiple stimuli, including telomere attrition, oncogenes, and cell stress.^[^ ^9^ ^]^ The primary hallmarks of cellular senescence are the increase in senescence‐associated β‐galactosidase (SA‐β‐Gal)‐positive cells and the upregulated expression of cyclin‐dependent kinase (CDK) inhibitors, such as p21, p53, and p16^INK4a^.^[^ ^10^ ^]^ Notably, the senescence‐associated secretory phenotype (SASP), comprising chemokines, pro‐inflammatory cytokines (IL‐1β, and IL‐6), and matrix metalloproteases (MMPs) can induce senescence‐like growth arrest.^[^ ^11^ ^]^ These phenotypic changes exacerbate the inflammatory microenvironment and ECM degradation within NPCs, ultimately accelerating IVDD.^[^ ^12^ ^]^ Despite the critical role of NPC senescence in IVDD, the underlying mechanisms and related signaling molecules require meticulous investigation. Angiotensin‐converting enzyme 2 (ACE2) is a carboxypeptidase that negatively regulates the renin–angiotensin system (RAS) and mediates the catabolism of angiotensin II (Ang II) to Ang‐(1–7).^[^ ^13^ ^]^ Although ACE2 and Ang‐(1–7) have been implicated in aging and senescence‐associated diseases in recent years,^[^ ^14^, ^15^ ^]^ the pathophysiological role of ACE2 in IVDD remains poorly understood. Our recent investigation revealed the presence of a local RAS in human IVD tissues, highlighting the detrimental effects of ACE/Ang II axis overactivation on NPC senescence and IVDD progression.^[^ ^16^ ^]^ However, to the best of our knowledge, the functions of ACE2 and its downstream signaling mechanisms during IVDD have not been determined.

In this study, we aimed to unveil the pathophysiological role of ACE2 in NPC senescence and IVDD progression. We first revealed that the expression of ACE2 correlates negatively with the degree of NPC senescence and IVDD at the histological, cellular, and animal levels. We then established *Ace2* knockout (*Ace2* ^−/y^) and *Ace2* overexpression (h*Ace2* ^OE^) mice and demonstrated that ACE2 deficiency increased the susceptibility of NPCs to senescence and exacerbated injury‐ or instability‐induced IVDD, whereas ACE2 overexpression counteracted these detrimental effects. An integrated analysis of single‐cell and bulk transcriptomics validated that ACE2 deficiency resulted in the activation of transforming growth factor beta‐2 (TGFβ2)/Smads signaling pathway and the transcription of *Serpine1*, a critical senescence activator, ultimately triggering NPC senescence and IVDD. Finally, we developed virus‐like nanovectors (VNs) loaded with small interfering RNA (siRNA) of *Serpine1*, a critical downstream signaling molecule of ACE2, for senescence‐retardation RNAi therapy of IVDD. Benefiting from their spiky surface nanotopology, VNs exhibited superior transfection efficiency compared to the commercial transfection reagent Lipofectamine 3000 (Lip3000). This nanomedical strategy could efficiently protect NPCs against senescence and intervene in IVDD by silencing Serpine1. Taken together, these findings highlight the critical role of ACE2 deficiency in NPCs senescence and its potential as a new molecular target for IVDD treatment.

## 2 Results

### 2.1 NPC Senescence is the Critical Contributor to IVDD

**Figure 1:**
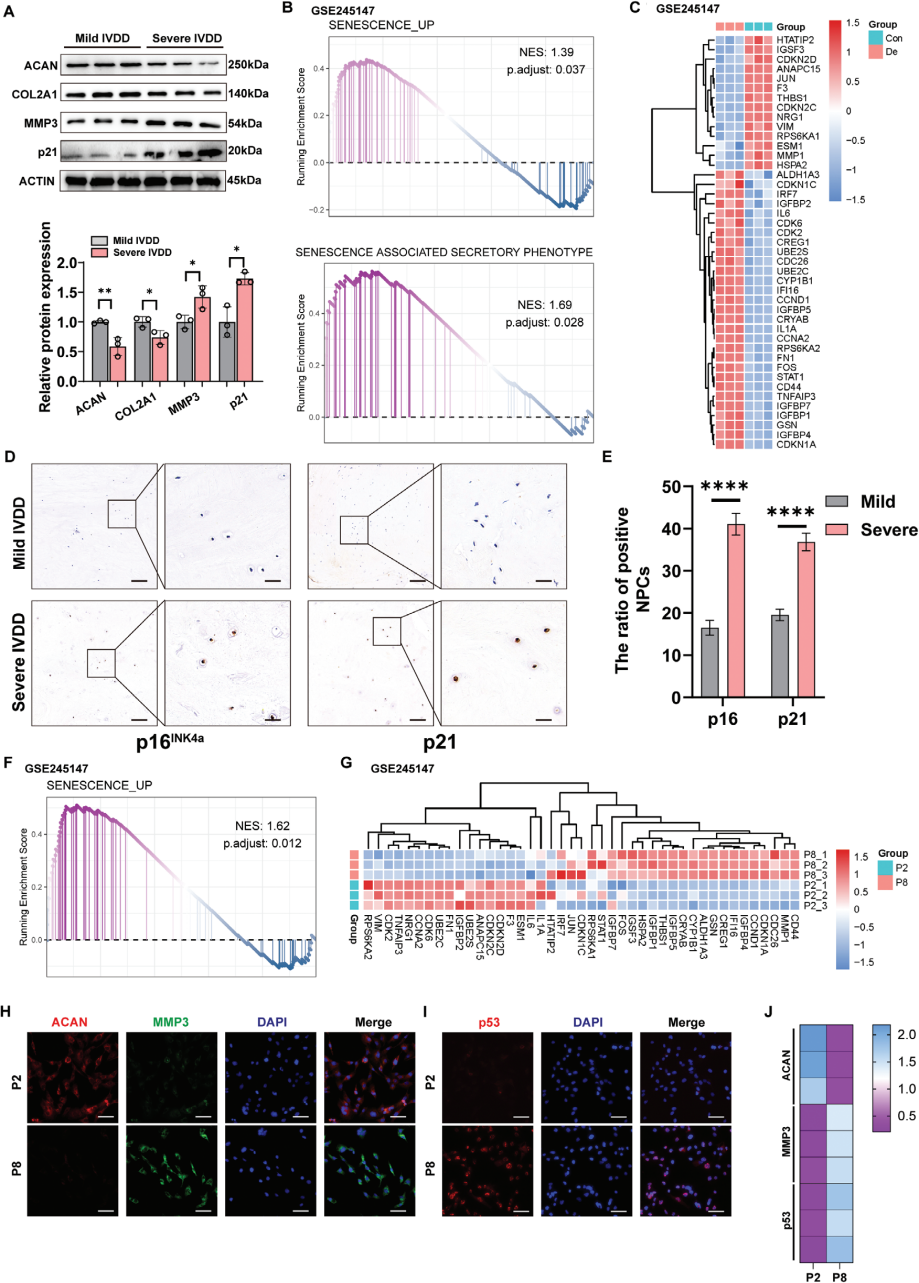
Senescence of NPCs during IVDD. A) Protein level analysis of ACAN, COL2A1, MMP3, and p21 in mildly and severely degenerated NP tissues by western blotting. ACTIN was used as a loading control. Statistical significance was determined using a two‐tailed unpaired Student's *t‐*test. B) GSEA analysis of sequencing data of control and degenerated NP tissue (GSE245147). The two‐sided Fisher's exact test with adjustments was used for multiple testing. C) Heatmap of representing different SASP expressions in control and degenerated NP tissue (GSE245147). D) IHC analysis of p16^INK4a^ and p21 expression in mildly and severely degenerated NP tissues (scale bar = 100 µm, 20 µm). E) Quantification of IHC results for p16^INK4a^ and p21 expression analyzed by a two‐tailed unpaired Student's *t*‐test. F) GSEA analysis of sequencing data NPCs at passage 2 (P2) and passage 8 (P8) (GSE245147). The two‐sided Fisher's exact test with adjustments was used for multiple testing. G) Heatmap of SASP expression in NPCs at P2 and P8 (GSE245147). H) IF analysis of ACAN and MMP3 expression in NPCs at P2 and P8 NPCs (scale bar = 50 µm). I) IF analysis of p53 expression in P2 NPCs and P8 (scale bar = 50 µm). J) Quantitative heat map of IF staining for ACAN, MMP3, and p53 in NPCs at P2 and P8. All data are presented as mean ± SD (*n =* 3). *p < 0.05, **p < 0.01, ***p < 0.001, and ****p < 0.0001. NPC: nucleus pulposus cell; NES: normalized enrichment score; GSEA: gene set enrichment analysis; IVDD: intervertebral disc degeneration; IF: immunofluorescence; ACAN: aggrecan; COL2A1: type 2 collagen; MMP3: matrix metalloproteinase‐3.

Excessive NPC senescence is a major pathological change during IVDD.^[^ ^17^ ^]^ To validate the role of NPC senescence in the progression of IVDD, we investigated the expression of senescence‐related molecules in degenerated NP tissues and NPCs. First, we found that samples from patients with severe IVDD showed upregulated protein expression of MMP3 and p21 and lower levels of Aggrecan (ACAN) and Type 2 Collagen (COL2A1) (**Figure** **1**A). We analyzed the RNA‐seq results for both normal and degenerated NP tissues (GSE245147). Abnormal activation of the cellular senescence‐related signaling pathway (NES = 1.39; *p* = 0.037) and the SASP (NES = 1.69; *p* = 0.028) was observed in degenerated NP tissues compared to that in normal tissues (Figure 1B). In addition, the degenerated NP tissues showed more differentially expressed genes (DEGs) related to the SASP (Figure 1C). Immunohistochemical (IHC) staining of patients’ NP samples further confirmed the upregulated expression of p21 and p16^INK4a^ in severely degenerated human NP tissue (Figure 1D,E).

NPCs at passages 2 (P2) and 8 (P8) were collected and subjected to RNA‐seq (GSE245147). Gene set enrichment analysis (GSEA) showed that NPCs underwent cellular aging and senescence with passaging (Figure 1F). Notably, NPCs at P8 exhibited activated senescence signaling pathways and expressed higher levels of SASP‐related genes than those at P2 (Figure 1F,G). This observation is consistent with the findings of DEGs in NPCs from degenerated NP tissues compared to NPCs from normal discs. Notably, the protein expression of MMP3 increased, whereas that of ACAN decreased in NPCs at P8 (Figure 1H,J). Furthermore, a markedly elevated expression of the senescence‐related marker p53 was detected in NPCs at P8, as evidenced by immunofluorescence (IF) staining and the quantitative results (Figure 1I,J). These findings suggest that NPCs senescence plays a pivotal role in the progression of IVDD.

### 2.2 ACE2 Expression is Negatively Correlated with IVDD Severity

To further explore the mechanism underlying NPC senescence in IVDD, we performed bioinformatics analysis of RNA‐seq data deposited in the Gene Expression Omnibus database (GSE15227 and GSE70362). The results of DEGs in both databases revealed a notable decrease in the expression of ACE2 in degenerated NP tissues (Figure S1A, Supporting Information). Kyoto Encyclopedia of Genes and Genomes (KEGG) analysis of DEGs indicated that the RAS was also activated in degenerated NP tissue (Figure S1B, Supporting Information). Furthermore, we evaluated the mRNA expression of *ACE2* in both human NP tissues and NPCs. Our analysis revealed that *ACE2* was downregulated in degenerated NP tissue (10 mildly degenerated and 20 severely degenerated) and in NPCs isolated from degenerated NP tissue (10 mildly degenerated and 10 severely degenerated) (Figure S1C,D, Supporting Information). All patients’ information is provided in the supplementary files (Table S1, Supporting Information). Safranin O and fast green (SOFG) staining were used to assess the degree of degeneration (Figure S1E, Supporting Information), obtaining results consistent with the Pfirrmann score based on T_2_‐weighed magnetic resonance imaging (MRI). IHC staining of NP tissues from patients demonstrated that the expression of ACE2 in severely degenerative NP tissues (Grade IV and V) was significantly lower than that in the moderate group (Grade II and III), whereas that of MMP3, a classical marker for IVDD, increased with the degree of degeneration (Figure S1E, Supporting Information). Single‐factor linear regression was conducted to explore the relationship between the percentage of ACE2‐ and MMP3‐positive NPCs and Pfirrmann grades in all 40 NP samples. The collected data revealed a positive correlation between MMP3 expression and IVDD Pfirrmann grades (*y*  =  12.06* × X – 4.681, *r*  =  0.695, *p* < 0.0001), and a negative correlation between ACE2 expression and IVDD Pfirrmann grades (*y*  =  −11.15* × X – 70.31, *r*  =  0.924, *p* < 0.0001) (Figure S1F,G, Supporting Information). Furthermore, we evaluated the expression of ACE2 in aging mice and a needle‐induced tail IVDD mouse model using hematoxylin and eosin (H&E), SOFG, and IF staining. As expected, a marked decrease in the expression of ACE2 in NP tissues from aging mice and the IVDD model was observed in the stained images (Figure S2, Supporting Information).

Previous studies reported that continuous passaging and inflammation contribute to NPCs senescence.^[^ ^18^ ^]^ To further examine the relationship between ACE2 and IVDD, we established a time‐lapse NPC passaging model by repeating each passage after doubling the number of NPCs. During continuous culture, the morphology of NPCs changed from fusiform at P0 to flattened at P8, as indicated by phalloidin staining (Figure S3A,B, Supporting Information). RT‐qPCR results demonstrated that continuous passaging resulted in the spontaneous degeneration of NPCs, characterized by decreased expression of *ACAN* and *COL2A1* and increased expression of *MMP3* and *MMP13* (Figure S3C, Supporting Information). Notably, both the gene and protein expression of ACE2 decreased with passage (Figure S3D–F, Supporting Information). Furthermore, we also established the IL‐1β‐induced NPCs degeneration model, with the results aligning with those of the NPCs passaging model (Figure S3G–L, Supporting Information). These data suggest that ACE2 deficiency is involved in NPC senescence.

### 2.3 ACE2 Deficiency Increases the Susceptibility of NPCs to Senescence and Aggravates IVDD

**Figure 2:**
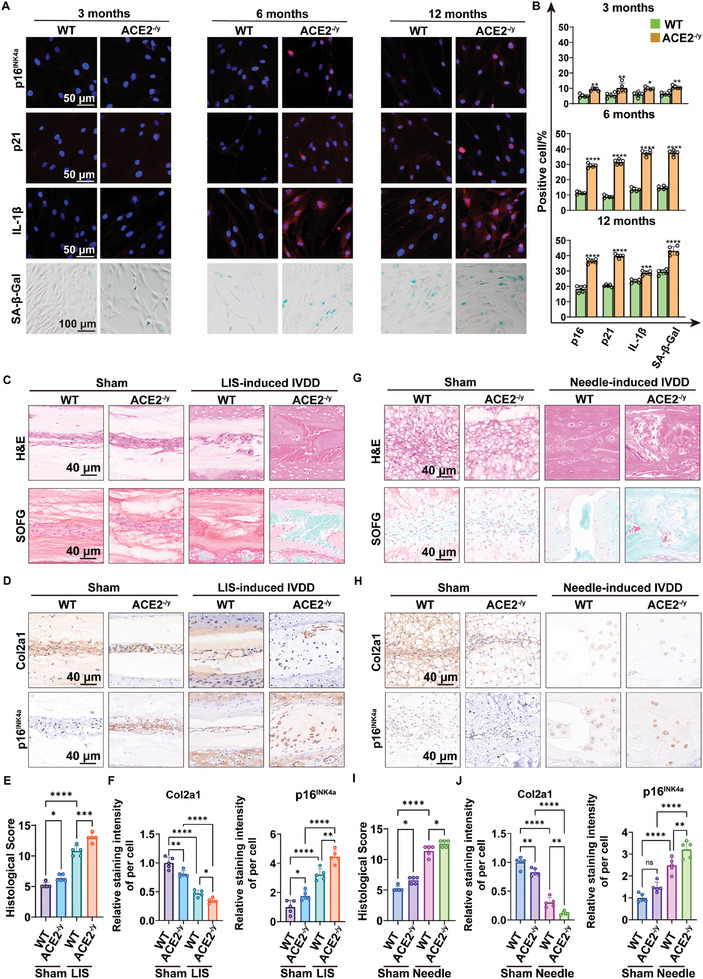
ACE2 deficiency increased the susceptibility of NPCs to senescence and aggravated IVDD. A) Representative images of immunofluorescence of p16^INK4a^, p21, IL‐1β, and SA‐β‐Gal staining in cultured NPCs from WT and *Ace2* ^−/y^ mice at 3, 6, and 12 months of age, respectively (scale bar = 50 µm, 100 µm). B) The quantification of IF results for p16^INK4a^, p21, and IL‐1β expression, and of SA‐β‐Gal staining, analyzed using a two‐tailed unpaired Student's *t*‐test. C) Representative images of H&E and SOFG staining of IVD tissue from WT and *Ace2* ^−/y^ mice with or without lumbar instability (scale bar = 40 µm). D) Representative images of IHC staining for COL2A1 and p16^INK4a^ of IVD tissue from WT and *Ace2* ^−/y^ mice with or without the surgery of lumbar instability (scale bar = 40 µm). E) Histological score of IVD tissue from WT and *Ace2* ^−/y^ mice with or without lumbar instability. F) The quantification of IHC results for COL2A1 and p16^INK4a^ of IVD tissue from WT and *Ace2* ^−/y^ mice with or without lumbar instability. G) Representative images of H&E and SOFG staining of IVD tissue from WT and *Ace2* ^−/y^ mice with or without the surgery of tail needling (scale bar = 40 µm). H) Representative images of IHC staining for COL2A1 and p16^INK4a^ of IVD tissue from WT and *Ace2* ^−/y^ mice with or without needling‐induced IVDD (scale bar = 40 µm). I) Histological score of IVD tissue from WT and *Ace2* ^−/y^ mice with or without needling‐induced IVDD. J) The quantification of IHC results for COL2A1 and p16^INK4a^ of IVD tissue from WT and *Ace2* ^−/y^ mice with or without needling‐induced IVDD. *p* values were determined by two‐way ANOVA followed by Tukey's post hoc test. All data are presented as mean ± SD (*n =* 5 per group). *p < 0.05, **p < 0.01, ***p < 0.001, and ****p < 0.0001. NPC; nucleus pulposus cell; IVDD: intervertebral disc degeneration; ACE2: angiotensin‐converting enzyme II; IF: immunofluorescence; H&E: Hematoxylin and eosin; SOFG: Safranin O/fast green; IHC: immunohistochemistry. LIS: lumbar instability.

Given the correlation between ACE2 deficiency and an increase in senescence‐related NPCs, we hypothesized that ACE2 may promote age‐ and injury‐induced NPC senescence and IVDD. To verify this hypothesis, we isolated NPCs from *Ace2* ^−/y^ mice and their wild‐type (WT) littermates at 3, 6, and 12 months of age, respectively, and cultured primary NPCs in vitro. IF staining suggested higher levels of the senescence phenotypes and SASP, including the expression of p16^INK4a^, p21, and IL‐1β in ACE2‐deficient NPCs (**Figure** **2**A,B). Moreover, the induction of SA‐β‐Gal was observed more in NPCs from *Ace2* ^−/y^ mice at the ages of 3, 6, and 12 months, respectively, compared to WT littermates (Figure 2A,B). In addition, we replated NPCs from *Ace2* ^−/y^ mice and their WT littermates at the age of 3 months and passaged them until P8. In contrast to those from WT littermates, NPCs at P8 from *Ace2* ^−/y^ mice showed lower mitochondrial membrane potential (ΔψM) (Figure S4A,B, Supporting Information). Enzyme‐linked immunosorbent assay (ELISA) indicated that ACE2 deficiency markedly increased the secretion of SASP factors including IL‐1β, IL‐6, IL‐13, MMP3, and MMP13, in both P2 and P8 NPCs from *Ace2* ^−/y^ mice (Figure S4C, Supporting Information). The results substantiate the detrimental effects of ACE2 deficiency on the dysfunction and senescence of NPCs.

Furthermore, we evaluated the pathological role of ACE2 in NPC senescence and IVDD using *Ace2* ^−/y^ mice (aged 3 months) and their WT littermates. Mice with lumbar instability (LIS)‐induced IVDD were included in this study. Histological analysis (H&E and SOFG staining) indicated that in the sham group, *Ace2* ^−/y^ mice showed greater loss of NP tissue, proteoglycan content, and number of NPCs than their WT littermates (Figure 2C,E; Figure S5A,B, Supporting Information). More severe and remarkable indicators, including a lower level of COL2A1 and a higher level of p16^INK4a^, were observed in the *Ace2* ^−/y^ mice in the LIS‐induced IVDD group (Figure 2D). The findings imply that *Ace2* ^−/y^ mice are susceptible to spontaneous IVDD affected by mechanical stress. We also established needle‐induced IVDD in the tail to mimic injury‐related stress and observed similar histological and molecular alterations in *Ace2* ^−/y^ mice (3 months old) compared with their WT littermates (Figure 2G–J; Figure S5C,D, Supporting Information). Collectively, our results suggest that ACE2 deficiency contributes to aging and aggravates mechano‐ and injury‐induced and IVDD.

### 2.4 ACE2 Overexpression Alleviates NPC Senescence and IVDD

As the main mediator of ACE2 function, Ang‐(1–7) was introduced to mimic the effects of ACE2 overexpression on NPC senescence.^[^ ^19^ ^]^ First, we investigated its biological effects on NPCs. Cell viability testing indicated that Ang‐(1–7) at a concentration of 10^−6^ m resulted in the strongest cell protective effect on H_2_O_2_‐treated (100 µM) NPCs (Figure S6A,B, Supporting Information). DCFH‐DA staining further demonstrated that Ang‐(1–7) could evidently decrease the intracellular reactive oxygen species in H_2_O_2_‐treated (100 µm) NPCs (Figure S6C, Supporting Information). Moreover, we cultured the primary NPCs with H_2_O_2_ (100 µm) in a time‐dependent manner to induce cellular senescence in vitro, with or without the administration of Ang‐(1–7) (10^−6^ m). IF staining suggested that NPCs treated with Ang‐(1–7) exhibited lower levels of senescence‐related factors, including γ2AX and p21, and the induction of SA‐β‐Gal‐positive NPCs, compared to untreated NPCs (Figure S7A,B, Supporting Information). In addition, Ang‐(1–7) restored the ΔψM of H_2_O_2_‐treated NPCs (Figure S7C, Supporting Information). ELISA assay indicated that the secretion of SASP in H_2_O_2_‐treated NPCs can be also alleviated by Ang‐(1–7) treatment (Figure S7D, Supporting Information). Thus, these observations certify the protection of ACE2/Ang‐(1–7) against NPC senescence.

To further evaluate the impact of ACE2 overexpression on NPC senescence and IVDD, we established CAG promoter‐driven homo‐ACE2‐transgenic (h*Ace2* ^OE^) mice characterized by enhanced ACE2 expression.^[^ ^20^ ^]^ The LIS‐related IVDD model was first established using h*Ace2* ^OE^ mice and WT littermates (aged 3 months). Staining images of H&E and SOFG and histological scoring indicated no significant differences in the volume of NP tissue and proteoglycan content between h*Ace2* ^OE^ mice and their WT littermates in the sham group (Figure S7E,G and S8A,B, Supporting Information). However, in the LIS‐induced IVDD group, h*Ace2* ^OE^ mice showed less severe degeneration‐related histological alterations, including improved NP tissue volume, high COL2A1 expression, and low expression of the senescence‐related marker p16^INK4a^ (Figures S7E–H and S8A,B, Supporting Information). Additionally, the needle‐induced IVDD model revealed similar histological and molecular alterations to that in the LIS‐induced IVDD model, indicating that *hAce2* ^OE^ mice exhibited higher tolerance to NPC senescence and IVDD response to injury stress (Figures S7I–L and S8C,D, Supporting Information). These findings demonstrate that enhancing the ACE2/Ang‐(1–7) axis can efficiently alleviate IVDD by mitigating NPC dysfunction and senescence.

### 2.5 ACE2 Deficiency Upregulates the Senescence‐related NPC Subcluster

We used the *Ace2* ^−/y^ mouse model to further evaluate the mechanistic association between ACE2 and NPCs senescence and IVDD. NP tissues from the tails of *Ace2* ^−/y^ mice (six months) and age‐matched WT littermates (*n =* 2 per group) were subjected to single‐cell RNA sequencing (scRNA‐seq) to determine cellular composition (Figure S9, Supporting Information). After quality control, we obtained a total of 13 231 cells from the NP tissue of *Ace2* ^−/y^ mice, and 12 891 cells from age‐matched WT littermates (Figure S10A, Supporting Information). Cells were grouped based on the expression of classical markers for annulus fibrosus (AF) cells (Chad, Vcan, and Cytl1),^[^ ^21^ ^]^ NPCs (Car3, T, and Lyst),^[^ ^22^ ^]^ macrophages (Lyz2, Cd74, and Rgs5),^[^ ^23^ ^]^ IFN‐response cells (Scd1, Ifitm3, and Atf3),^[^ ^24^ ^]^ and cycling cells (Top2a, Mki67, and Ube2c), as well as the cell ratio of all the identified cell clusters (Figure S10B–D, Supporting Information).

**Figure 3:**
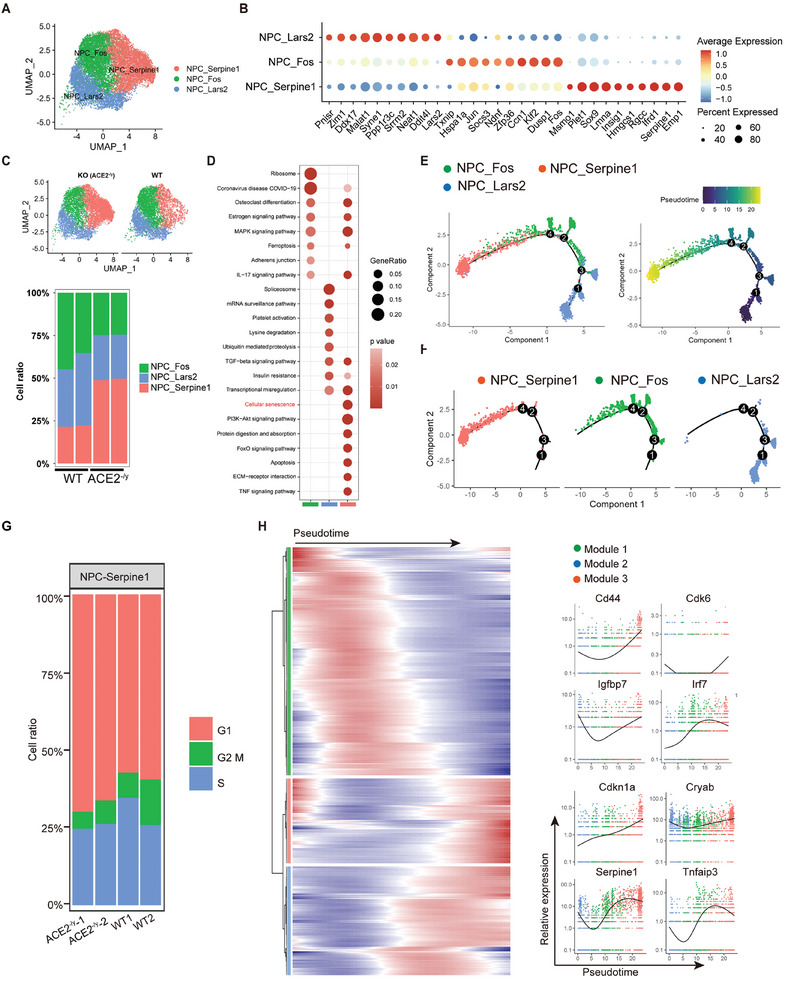
NPC‐Serpine1 cluster was identified as a new NPC cluster of cellular senescence in ACE2‐deficient mice. A) Visualization of clustering by uniform manifold approximation and projection (UMAP) plot of NPC clusters in IVD samples from WT and *Ace2* ^−/y^ mice (*n =* 2 per group). B) Dot plot of highest DEGs for each major NPC type (right axis). Dot color intensity represents the z‐score of expression values, and dot size represents the percent of cells with at least one UMI detected per gene. C) UMAP plot showing the proportion of the three NPC subtypes in WT and *Ace2* ^−/y^ mice (*n =* 2 per group). D) KEGG analysis of the three NPC subtypes in WT and *Ace2* ^−/y^ mice (*n =* 2 per group). E,F) Monocle pseudotime trajectory axis reveals the progression of the three NPC subclusters. G) Cell cycle of NPC‐Serpine1 cluster in WT and Ace2^−/y^ mice (*n =* 2 per group). H) Expression of selected DEGs for each branch. The senescence‐related genes were mapped in the gene modules in pseudotime analyzed with Monocle and colored in developmental stages. NPC: Nucleus pulposus cell; KEGG: Kyoto encyclopedia of genes and genomes; DEG: Differentially expressed genes; IVD: Intervertebral disc.

To identify downstream molecules, we analyzed NPCs subpopulations in NP tissues from *Ace2* ^−/y^ mice and their WT littermates using Seurat unsupervised clustering in the R package.^[^ ^25^ ^]^ Three distinct NPC clusters were identified based on their characteristic marker genes: NPC‐Serpine1, NPC‐Fos, and NPC‐Lars2 (**Figure** **3**A,B). Notably, the proportion of NPC‐Fos and NPC‐Lars2 decreased, whereas that of NPC‐Serpine1 increased in *Ace2* ^−/y^ mice compared to control WT mice (Figure 3C). KEGG analysis was conducted to gain a deeper insight into the function of different NPC clusters. These data indicated that NPC‐Serpine1 was associated with cellular senescence, apoptosis, and the IL‐17 signaling pathway. NPC‐Fos exhibited enrichment in the ribosome, coronavirus disease COVID‐2019, and estrogen signaling pathways, while NPC‐Lars2 was characterized by the spliceosome, mRNA surveillance pathway, and platelet activation (Figure 3D).

Subsequently, we used Monocle2 to reconstruct the differentiation trajectory of the NPCs lineage in pseudotime. The overall differentiation trajectory of disc degeneration was revealed, starting from NPC‐Lars2 and branching toward NPC‐Fos and NPC‐Serpine1 (Figure 3E). By analyzing each NPC subpopulation in the pseudo‐time trajectory, it was found that NPC‐Lars2 was at the starting position, whereas NPC‐Serpine1 was at the terminal disease stage (Figure 3F). Notably, the cell cycle results showed that the proliferative ability of NPC‐Serpine1 was decreased in *Ace2* ^−/y^ mice, indicating cell cycle inhibition in ACE2‐deficient NPCs (Figure 3G). To explore senescence‐related gene expression dynamics along pseudo‐time trajectories, we explored DEGs that constantly increased or decreased during IVDD and grouped them according to their expression patterns. We identified three grouped gene modules based on their differences (Figure 3H). Module 1 was upregulated at an earlier stage, whereas modules 2 and 3 were upregulated at a later stage of the disease. Notably, senescence‐related genes, including *Cryab, Irf7*, *Igfbp7*, *Cdk6*, *Tnfaip3*, *Cdkn1a*, *Serpine1*, and *Cd44*, showed an upward trend during NPC differentiation. Furthermore, the analysis of transcription factors suggested that NPC‐Serpine1 expressed more inflammation‐related genes, including Nfkb2, Rel, and Jdp2, which are involved in the production of SASP factors (Figure S11, Supporting Information).^[^ ^26^ ^]^ Taken together, these findings indicate that NPC‐Serpine1 may represent a critical senescence‐related sub‐cluster that contributes to NPC dysfunction and IVDD in the absence of ACE2.

### 2.6 ACE2 Deficiency Contributes to NPC Senescence via TGFβ2/Smads‐Mediated Transcription of *Serpine1*

**Figure 4:**
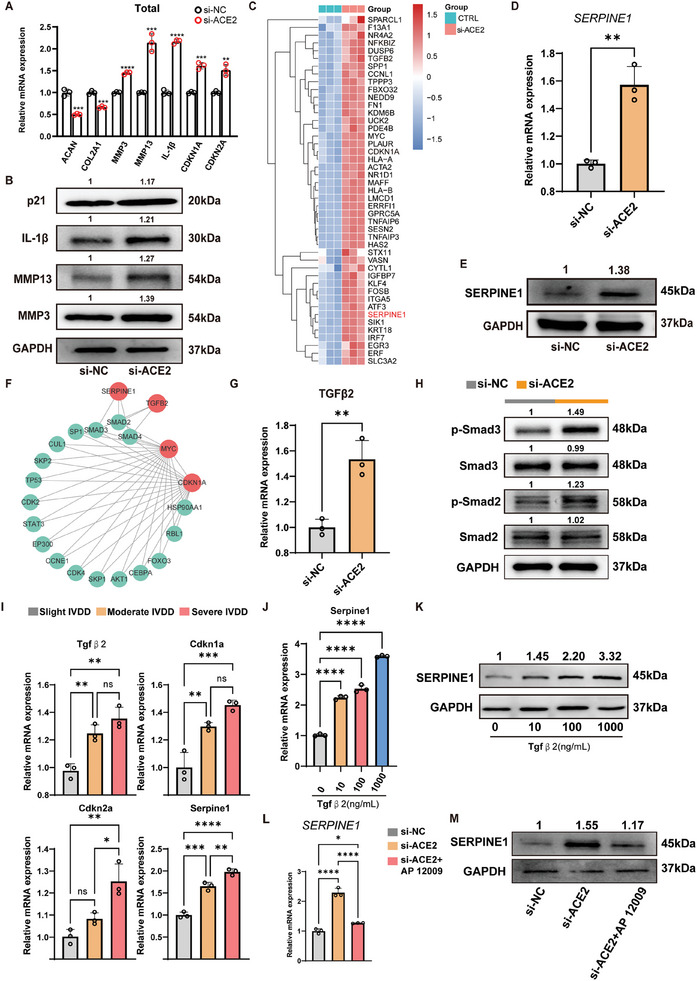
ACE2 deficiency contributes to NPC senescence via TGFβ2/Smads‐mediated transcription of *SERPINE1*. A) RT‐qPCR analysis of degeneration‐ and senescence‐related genes in human NPCs with or without silencing ACE2 (*n =* 3 per group). Statistical significance was determined using a two‐tailed unpaired Student's *t*‐test. B) Western blotting analysis of degeneration‐ and senescence‐related genes in human NPCs with or without silencing ACE2. C) Bulk RNA sequencing of DEGs in human NPCs with or without silencing ACE2 (*n =* 3 per group). D) RT‐qPCR analysis of *SERPINE1* expression in human NPCs with or without silencing ACE2 (*n =* 3 per group). Statistical significance was determined using a two‐tailed unpaired Student's *t*‐test. E) Western blot analysis of SERPINE1 expression in human NPCs with or without silencing ACE2. F) Protein–protein interaction analysis of the DEGs related to Serpine1 based on the bulk RNA sequencing. G) RT‐qPCR analysis of *TGFβ2* expression in human NPCs with or without silencing ACE2 (*n =* 3 per group). Statistical significance was determined using a two‐tailed unpaired Student's t‐test. H) Western blotting analysis of Smad2, Smad3, p‐Smad2, and p‐Smad3 expression in human NPCs with or without silencing ACE2. GADPH was used as the reference protein. I) RT‐qPCR analysis of *TGFβ2, CDKN1A, CDKN2A, and SERPINE1* expression in human NP tissue with different degeneration degrees (*n =* 3 per group). Two‐way analysis of variance (ANOVA) followed by Tukey's post hoc test was used to determine statistical differences. J) RT‐qPCR analysis of *SERPINE1* expression in human NPCs treated by TGFβ2 in a dose‐dependent manner (*n =* 3 per group). Two‐way analysis of variance (ANOVA) with Tukey's post hoc test was used to determine statistical differences. K) Western blotting analysis of Serpine1 expression in human NPCs treated by TGFβ2 in a dose‐dependent manner. L) RT‐qPCR analysis of *SERPINE1* expression in *ACE2*‐knocking down NPCs with or without blocking TGFβ2 using AP 12 009 (*n =* 3 per group). Two‐way analysis of variance (ANOVA) with Tukey's post hoc test was used to determine statistical differences. M) Western blotting analysis of SERPINE1 expression in ACE2‐knocking down NPCs with or without blocking TGFβ2 using AP 12 009. All data are presented as the mean ± SD. *p < 0.05, **p < 0.01, ***p < 0.001, and ****p < 0.0001. NPC: nucleus pulposus cell; ACE2: angiotensin‐converting enzyme 2; Serpine1: Serpin family E member 1; CDKN1A: cyclin‐dependent kinase inhibitor 1A; CDKN2A: cyclin‐dependent kinase inhibitor 2A; TGFβ2: transforming growth factor beta 2.

To identify potential molecular targets for gene therapy, we used *ACE2* siRNA with optimized transfection efficiency to establish NPCs with knocked down *ACE2* gene, as demonstrated by RT‐qPCR (Figure S12, Supporting Information). As expected, knocking down *ACE2* resulted in the downregulation of *ACAN* and *COL2A1* and upregulation of senescence‐related genes, including *MMP3*, *MMP13*, *IL‐1β*, *CDKN1A*, and *CDKN2A* (**Figure** **4**A). Western blotting analysis further confirmed the pro‐senescent effects of ACE2 deficiency (Figure 4B). In addition, we found that the Ang‐(1–7)–MasR axis significantly restored *ACE2* deficiency‐induced NPC senescence and degeneration (Figure S13A, Supporting Information). Subsequently, we performed RNA‐seq analysis in normal and *ACE2*‐knocked down NPCs and crossed the upregulated DEGs with the scRNA‐seq results, ultimately identifying 45 DEGs (Figure S14, Supporting Information). Surprisingly, we found that the expression of *SERPINE1* was upregulated after *ACE2* knockdown, which was consistent with the results of scRNA‐seq (Figure 4C). This result was further confirmed by RT‐qPCR, Western blotting analysis, and IF staining at both the mRNA and protein levels (Figure 4D,E; Figure S15A,B, Supporting Information). Gene Ontology analysis indicated that *SERPINE1* was closely related to cellular senescence (Figure S16, Supporting Information). Thereafter, we used siRNA targeting *Serpine1* to investigate whether ACE2 regulated NPC senescence via Serpine1 (Figure S17, Supporting Information). The antisenescence effect of silencing *Serpine1* was evaluated by western blotting assay that inhibiting *SERPINE1* expression alleviated ACE2‐deficiency‐induced expression of senescent markers (Figure S18A, Supporting Information). Notably, the inhibition of *Serpine1* also ameliorated H_2_O_2_‐mediated NPC senescence, as shown by the results of the expression of senescence‐related proteins (Figure S18B, Supporting Information). Therefore, it can be concluded that ACE2 deficiency contributes to cellular senescence by elevating the expression of Serpine1.

Then, we performed protein–protein interaction (PPI) analysis and identified that the TGFβ2/SMADs signaling pathway may mediate the regulatory of ACE2 on Serpine1 (Figure 4F). GSEA analysis further revealed that ACE2 deficiency contributed to the activation of the TGFβ signaling pathway (Figure S19A, Supporting Information). In addition, it was shown that silencing *ACE2* resulted in increased expression of TGFβ2 (Figure 4G). Western blotting and IF staining results indicated that silencing *ACE2* significantly increased the phosphorylation of Smad2 and Smad3, which mediates the bioeffects of the TGFβ2 signaling pathway (Figure 4H; Figure S19B,C, Supporting Information). The findings suggest that *ACE2* regulates the expression of *Serpine1* via the TGFβ2/Smad2/3 signaling pathway. TGFβ is a pleiotropic cytokine that regulates a myriad of cellular functions, including NP microenvironment.^[^ ^27^ ^]^ However, the role of TGFβ2 in the progression of IVDD remains elusive. Here, we unveiled the role of the Ang‐(1–7)/MasR axis in mediating the ACE2‐deficiency‐induced expression of TGFβ2 (Figure S13B, Supporting Information). Additionally, we analyzed the expression of TGFβ2 in NP tissues with different degeneration degrees. A higher level of *TGFβ2* mRNA expression was observed in severe IVDD (Figure 4I). In addition, the mRNA expression of *CDKN1A*, *CDKN2A*, and *SERPINE1* also increased depending on the degree of degeneration, further confirming the relationship between *TGFβ2* and NPC senescence. Notably, the treatment with recombinational TGFβ2 protein dose‐dependently promoted the expression of *SERPINE1* in NPCs both at mRNA and protein levels (Figure 4J,K). Conversely, repressing *TGFβ2* using AP12009, an anti‐sense oligodeoxynucleotide specifically targeting TGFβ2, reduced the expression of *SERPINE1* induced by ACE2 deficiency (Figure 4L,M). Collectively, these observations indicate that TGFβ2/Smads pathway‐mediated transcription of *SERPINE1* serves as a pivotal downstream mechanism for ACE2 deficiency‐mediated NPC senescence.

### 2.7 Nanotopology‐Enhanced Transfection Efficiency of Virus‐Like Nanovectors

**Figure 5:**
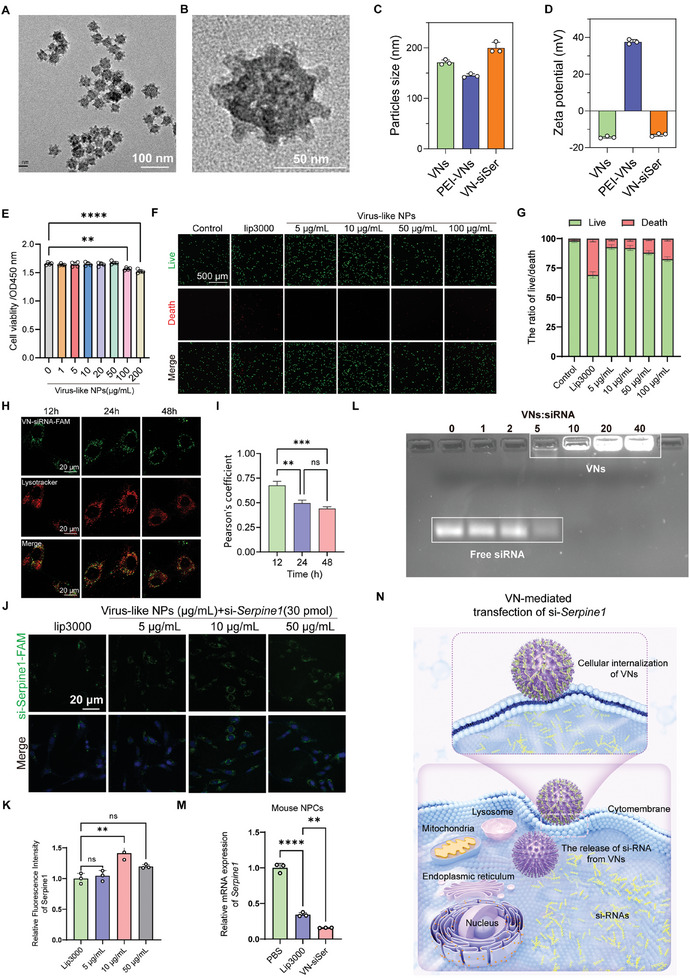
The characteristics and transfection efficiency of virus‐like mesoporous silica nanoparticles (VNs). A) TEM image of VNs (scale bar = 100 nm). B) Enlarged image of urchin‐like ceria nanoparticles (scale bar = 50 nm). C) Hydrodynamic size of VNs and VN‐siSer compared using a two‐tailed unpaired Student's *t*‐test. D) The Zeta potential of VNs and VN‐siSer compared using a two‐tailed unpaired Student's *t*‐test. E) Cell viability assay of NPCs treated with VNs of different concentrations (*n =* 4 per group) analyzed using a two‐tailed unpaired Student's *t*‐test. F) Live–dead staining of NPCs incubated with Lip3000 and VNs with different concentrations (scale bar = 500 µm). G) Quantitative results of Live/dead staining of NPCs. H) Confocal microscopy images showing the intracellular uptake of FAM‐labelled VN‐siRNA at 12 h, 24 h, and 48 h. Lysosomes were stained with LysoTracker (Red) (Scale bar = 20 µm). I) Temporal evaluation of Pearson's coefficient for VN‐siSers. P values were determined by two‐way ANOVA with Tukey's post hoc test. J) Transfection of FAM‐labeled si‐*Serpine1* into NPCs cocultured with different concentrations of VNs (5, 10, and 50 µg mL^−1^) or Lip3000‐siSer (scale bar = 20 µm). K) Quantitative results of the transfection of fluorescence in panel H. *p* values were determined by two‐way ANOVA followed by Tukey's post hoc test. L) Agarose gel assay of VN/si‐Serpine1 complexes with different weight ratios. M) The expression of mouse *Serpine1* in mouse NPCs treated with VN‐siSer or Lip3000‐siSer by RT‐qPCR. *p* values were determined by two‐way ANOVA followed by Tukey's post hoc test. N) The schematic diagram of the transfection of si‐*Serpine1* into NPCs by VNs. *p < 0.05, **p < 0.01, ***p < 0.001, and ****p < 0.0001. TEM: Transmission electron microscope; Serpine1: Serpin family E member 1.

Viruses have demonstrated remarkable cellular invasion properties owing to their rough surface, which consists of spike proteins that can strongly bind to cell membranes during the invasion process.^[^ ^28^ ^]^ Inspired by the distinctive surface topology of viruses, we engineered virus‐like mesoporous silica nanoparticles (MSNs) and utilized them as unique nanovectors with rough spiky tubular surfaces to establish a nanotopology‐enhanced siRNA delivery system.^[^ ^29^ ^]^ First, virus‐like MSNs were synthesized via a single‐micelle epitaxial growth approach using a low‐concentration surfactant in an oil–water biphasic system.^[^ ^29^ ^]^ VNs were modified by using a cationic polymer polyethylenimine (PEI). The thermal gravimetric analysis (TGA) carried out in the nitrogen atmosphere confirmed that the modified VNs lost 23.6% mass compared to the bare VNs between 100 and 800 °C, which was attributed to the decomposition of PEI (Figure S20, Supporting Information). Subsequently, Serpine siRNA (siSer) was absorbed onto VNs via electrostatic interactions. Transmission electron microscopy (TEM) images showed the virus‐like morphology of PEI‐modified MSNs (**Figure** **5**A,B). The hydrodynamic diameter of VN‐siSer was slightly larger than that of bare VN (Figure 5C). Correspondingly, the absolute value of the zeta potential for VN‐siSer decreased, implying the successful absorption of siSer (Figure 5D). We conducted in vitro experiments to evaluate the cytotoxicity and biocompatibility of the VNs. CCK‐8 results showed that VNs had negligible toxicity toward NPCs up to a concentration of 200 µg mL^−1^ (Figure 5E). Live/dead staining showed only weak red fluorescence representing dead NPCs during treatment with VN within concentrations from to 5–100 µg mL^−1^, whereas noticeable red spots appeared in the group of NPCs treated with Lip3000 (3 µL, L3000075, Lipofectamine 3000, ThermoFisher Scientific) (Figure 5F,G).

Lysosome‐mediated endocytosis is an important mechanism by which nanomaterials enter cells.^[^ ^30^ ^]^ In addition, the normal functioning of nanoparticles relies on their efficient uptake by cells and timely escape from endosomes. Therefore, the internalization pathway of fluorescein amidite (FAM)‐labeled VN‐siRNA into NPCs was evaluated. We selected inhibitors of clathrin‐mediated endocytosis (chlorpromazine), caveolae‐mediated endocytosis (genistein), and micropinocytosis (amiloride). The results demonstrated that cellular uptake of VN‐siRNA‐FAM in NPCs was inhibited by these inhibitors (Figure S21A–C, Supporting Information), especially genistein and amiloride, indicating that caveolae‐mediated endocytosis and micropinocytosis are the predominant pathways that mediate the cellular internalization of VN‐siRNA‐FAM into NPCs. These observations were consistent with a previous study.^[^ ^29^ ^]^ Owing to the specific nanotopology of the developed nanovectors, it is speculated that both the conventional energy‐consuming process and the biomechanical effects contribute to the internalization of VN‐siRNA. To evaluate the endosomal escape of VN‐siRNA after cellular uptake, we examined the subcellular localization of nanovectors in NPCs using fluorescence imaging. The results showed that VN‐siRNA‐FAM demonstrated significant endosomal escape after 12 h of incubation, with only a small number of yellow fluorescence spots observed, indicating limited colocalization of LysoTracker Red and FAM‐labeled VN‐siRNA nanoparticles (Figure 5H,I). By 24 h, VN‐siRNA‐FAM achieved more efficient escape, as evidenced by the distinct separation of the red and green fluorescence. This successful lysosomal escape was further maintained and observed until 48 h, highlighting the sustained escape capability of VN‐siRNA‐FAM. Subsequently, the transfection efficiency of VNs was compared to that of Lip3000 using siRNA from the negative control (si‐NC) labeled with FAM as a marker. Fluorescent images showed that VN‐siNC possessed a higher transfection efficiency than Lip3000 (Figure S22A,B, Supporting Information).

SERPINE1, also known as plasminogen activator inhibitor 1 (PAI‐1), has been shown to promote cellular senescence.^[^ ^31^ ^]^ Given that the aforementioned findings underscore the pivotal role of Serpine1 as a downstream regulator of senescence, we explored the therapeutic potential of modulating the expression *SERPINE1* in NPC senescence. To examine the optimal concentration of VNs delivering si‐*SERPINE1* and the siRNA encapsulation efficiency of the VNs. Si‐*SERPINE1* labeled with FAM was mixed with different concentrations of VNs (5, 10, and 50 µg mL^−1^) and Lip3000 (3 µL) and then the mixture was added onto the culture medium. It was found that VNs could effectively deliver si‐*SERPINE1* into NPCs at a concentration of 10 µg mL^−1^, superior to that of Lip3000 (Figure 5J,K). Thus, 10 µg mL^−1^ was used as the optimal concentration in following transfection experiments. In addition, we evaluated the siRNA encapsulation efficiency of the VNs, and a gel retardation assay was performed. As shown in Figure 5L, si‐*SERPINE1* was fully loaded onto VNs with a weight ratio of VNs to si‐*SERPINE1* of 10:1 (Figure 5L).

Overall, these results validated the high efficiency of the developed VNs in silencing the transcriptional expression of mouse *Serpine1*, surpassing that of commercial Lip3000 (Figure 5M). Collectively, VNs can be used as an effective delivery system to transfect siRNA and its superior performance is attributed to the virus‐mimicking surface topology of the VNs (Figure 5N).^[^ ^32^ ^]^

### 2.8 Inhibiting *Serpine1* Using VN‐siSer Alleviates Senescence and the SASP in *Ace2*‐Deficient NPCs

**Figure 6:**
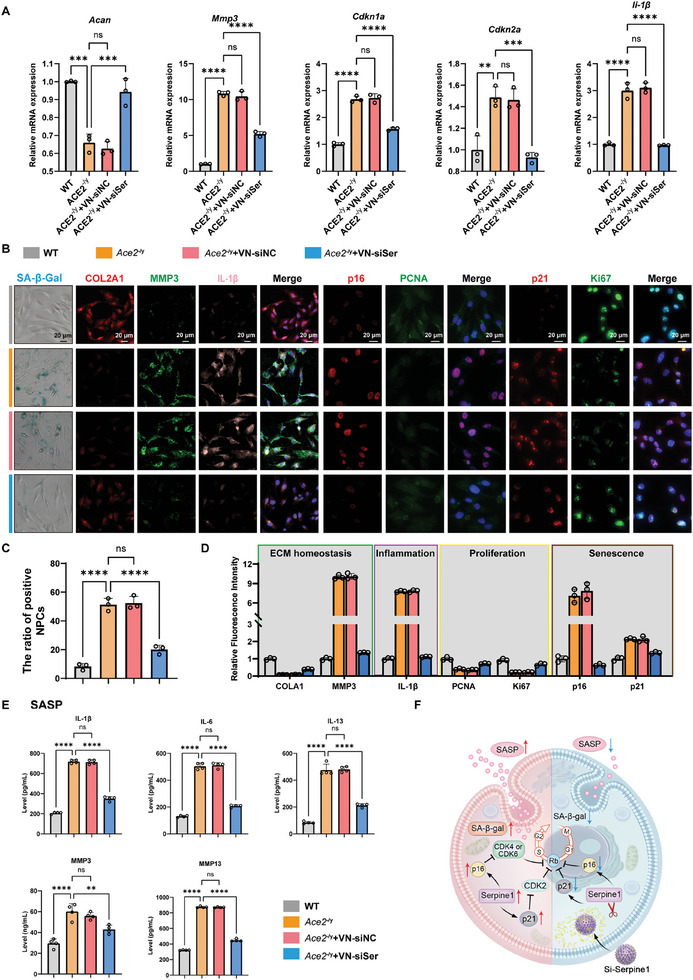
Inhibiting Serpine1 using VN‐siSer alleviated senescence and SASP in *Ace2*‐deficient mouse NPCs. A) The expression of degeneration‐ and senescence‐related genes (*Acan*, *Mmp3*, *Cdkn1a*, *Cdkn2a*, and *Il‐1β*) in *Ace2*‐deficient NPCs treated with VN‐siSer by RT‐qPCR. B) Representative images of immunofluorescence of COL2A1, MMP3, IL‐1β, p16, PCNA, p21, Ki67, and SA‐β‐Gal staining in *Ace2*‐deficient NPCs with or without VN‐siSer (scale bar = 20 µm). C) Quantitative results of SA‐β‐Gal staining. D) Quantitative results of immunofluorescence of COL2A1, MMP3, IL‐1β, p16, PCNA, p21, and Ki67 in *ACE2*‐deficient NPCs with or without VN‐siSer (*n =* 3 per group). E) ELISA results of the SASP‐related cytokines (IL‐1β, IL‐6, IL‐13, MMP3, and MMP13) content in the supernatant from in *Ace2*‐deficient NPCs with or without VN‐siSer (*n =* 3 per group) analyzed using a two‐tailed unpaired Student's *t* test. F) A schematic diagram of inhibition of *Serpine1* using VN‐siSer demonstrating alleviation of senescence and changes in the SASP in *Ace2*‐deficient NPCs. *p* values were determined by two‐way ANOVA followed by Tukey's post hoc test. All data are presented as mean ± SD. *p < 0.05, **p < 0.01, ***p < 0.001, and ****p < 0.0001. SERPINE1: Serpin family E member 1; NPC: nucleus pulposus cell; COL2A1: Type II collagen; MMP3: Metal matrix‐degrading enzyme 3; IL‐1β: Interleukin1β; PCNA: Proliferating cell nuclear antigen; SASP: senescence‐associated secretion phenotype; VN: virus‐like nanoparticle; ELISA: enzyme‐linked immunosorbent assay.

Next, we explored the therapeutic effects of *Serpine1* silencing on *Ace2* deficiency‐related mouse NPCs, and WT and *Ace2* ^−/^ *^y^* mice aged 12 months were used to study senescence. We selected an optimized siRNA targeting mouse *Serpine1* with the best transfection efficiency for in vivo treatment (Figure S23, Supporting Information). RT‐qPCR analysis revealed that *Ace2* deficiency decreased the expression of *ACAN* and increased the expression of senescence‐related cytokines, including *Mmp3*, *Cdkn1a*, *Cdkn2a*, and *Il‐1β* (**Figure** **6**A). In contrast, inhibiting the expression of *Serpine1* using VN‐siSer dramatically ameliorated these detrimental effects. Compared with the *Ace2*‐deficient group, treatment with VN‐siSer also upregulated the expression levels of COL2A1 and downregulated the expression of MMP3, indicating retention of the NPC phenotype (Figure 6B‐D). Furthermore, SA‐β‐Gal and IF staining images showed that VN‐siSer significantly decreased the ratio of senescent NPC, reduced the expressions of p16^INK4a^, p21, and IL‐1β, but increased the expression of PCNA and Ki67 (Figure 6B–D). These observations suggest the alleviation of senescence and recovery of the self‐repair capacity in NPCs by VN‐mediated siRNA treatment. In addition, we evaluated the SASP of NPCs in different groups. The results showed that *Ace2*‐deficient NPCs secreted large amounts of SASP components, including inflammatory molecules (IL‐1β, IL‐6, and IL‐13) and degradative enzymes (MMP3 and MMP13), which were markedly inhibited by administration of VN‐siSer (Figure 6E). Hence, by specifically suppressing the expression of *Serpine1*, VN‐siSer effectively inhibited the occurrence of downstream senescence‐associated events, thereby relieving NPC senescence induced by *Ace2* deficiency (Figure 6F).

Furthermore, we evaluated whether VN‐siSer exerts therapeutic effects by protecting NPCs from WT mice against H_2_O_2_‐triggered senescence. Similar to the results observed in *Ace2*‐deficient NPCs, *Serpine1* silencing by VN‐siSer markedly reduced the expression of SA‐β‐Gal in NPCs treated with H_2_O_2_ (Figure S24A,B, Supporting Information). Additionally, IF staining revealed downregulation in the expression of degeneration‐ (ACAN) and senescence‐ (MMP3 and p16^INK4a^) related biomarkers in H_2_O_2_‐treated NPCs following VN‐siSer treatment, suggesting its therapeutic effect in rescuing ROS‐induced NPC senescence (Figure S24C,D, Supporting Information). Collectively, the inhibition of *SERPINE1* by VN‐siSer treatment alleviated senescence and reversed the SASP in both *Ace2*‐deficient and H_2_O_2_‐treated NPCs, which is expected to facilitate the self‐repair process of NPCs after damage.

### 2.9 VN‐siSer Rescues NP Tissue Senescence and Degeneration in Injury‐Induced IVDD

**Figure 7:**
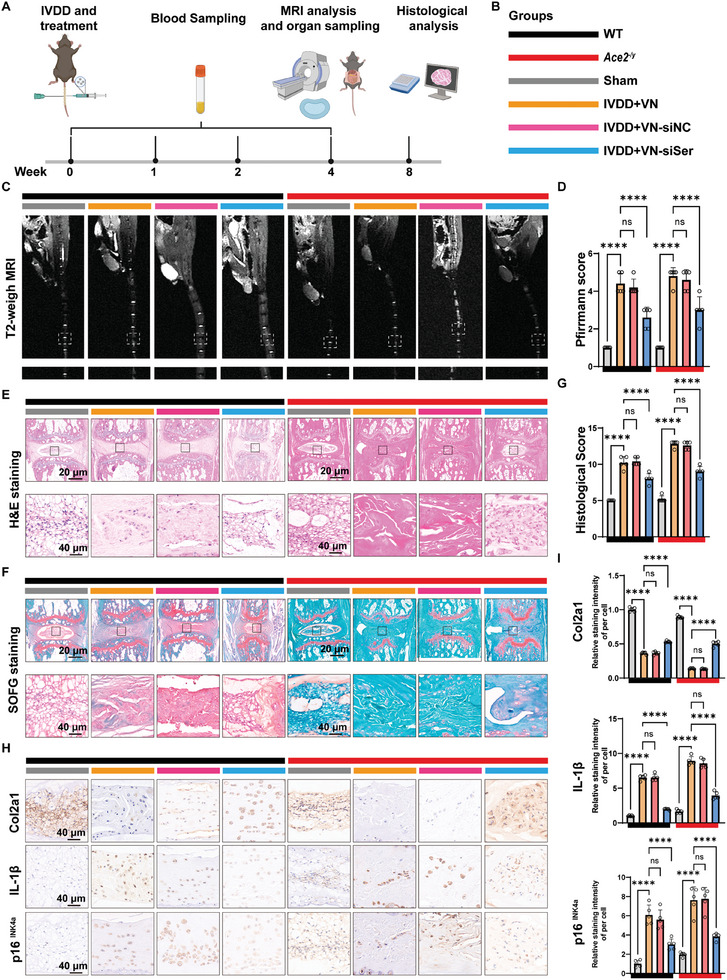
VN‐siSer can effectively alleviate NP tissue senescence and degeneration in mice with needle‐induced IVDD. (A and B) Schematic illustration of the experimental setup. (C and D) T2‐weighed MRI images and Pfirrmann grade analysis of WT and *Ace2* ^−/y^ mice with or without needling injury or the administration of VN‐siSer. E) Representative images of H&E staining of IVD tissue from WT and *Ace2* ^−/y^ mice with or without needling injury or the administration of VN‐siSer (scale bar = 20 µm, 40 µm). F) Representative images of SOFG staining of IVD tissue from WT and *Ace2* ^−/y^ mice with or without needling injury or the administration of VN‐siSer (scale bar = 20 µm, 40 µm). G) Histological score of IVD tissue from WT and *Ace2* ^−/y^ mice with or without needling injury or the administration of VN‐siSer (*n =* 5 per group) H) Representative images of IHC staining for COL2A1, IL‐1β, and p16^INK4a^ of IVD tissue from WT and *Ace2* ^−/y^ mice with or without needling injury or the administration of VN‐siSer (scale bar = 20 µm, 40 µm). I) The quantification of IHC staining for COL2A1, IL‐1β, and p16^INK4a^ of IVD tissue from WT and *Ace2* ^−/y^ mice with or without needling injury or the administration of VN‐siSer (*n =* 5 per group). *p* values were determined by two‐way ANOVA followed by Tukey's post hoc test. All data are presented as mean ± SD. *p* values were determined by two‐way ANOVA followed by Tukey's post hoc test. *p < 0.05, **p < 0.01, ***p < 0.001, and ****p < 0.0001. IVD: Intervertebral disc; NPC: nucleus pulposus cell; COL2A1: Type II collagen; MMP3: Metal matrix‐degrading enzyme 3; IL‐1β: Interleukin1β.

Next, we established needle‐induced IVDD in mice to investigate the in vivo therapeutic effect of VN‐siSer (**Figure** **7**A). To confirm the protective effects of VN‐siSer on NPCs against degeneration and senescence, we established the IVDD model in WT and *Ace2* ^−/y^ mouse, respectively (Figure 7B). The IVDD mice received weekly injections (once a week) of VNs, VN‐siNC, and VN‐siSer. Four weeks after the IVDD surgery, all mice were euthanized for MRI and histological evaluation. MRI can reliably determine variations in the water content in the disc, with increased T_2_‐weighted signals indicating higher NP water content.^[^ ^33^ ^]^ In WT mice, needle puncture resulted in obvious water loss (low and even disappearing T_2_‐weighted signals) in the NP tissue, whereas the MRI signals of the NP tissue in the VN‐siSer group recovered to a greater extent (Figure 7C). The Pfirrmann scores among the different groups of WT mice showed a similar trend (Figure 7D). Similarly, in *Ace2* ^−/y^ mice, the administration of VN‐siSer restored the signals of NP tissue and Pfirrmann scores compared to the IVDD + VNs and IVDD + VN‐siNC groups. Thus, imaging evaluation results demonstrate that inhibiting *Serpine1* could alleviate needle‐mediated IVDD in both WT and *Ace2* ^−/y^ mice.

Furthermore, we evaluated the histological changes in mouse tails in all groups.^[^ ^17^ ^]^ H&E and SOFG staining showed that in the sham groups, the intervertebral disc remained intact, with the NP tissue distinctly demarcated from the surrounding tissue, and that the annulus fibrosus (AF) arranged in an ordered manner (Figure 7E–G). Conversely, needle puncture significantly decreased intervertebral height, which was accompanied by disorganized AF and a disrupted matrix structure. Notably, VN‐siSer treatment maintained the NP and AF tissues in a relatively normal structure in both WT and *Ace2* ^−/y^ mice (Figure 7E–G). The decreased expression of COL2A1 and increased expression of p16^INK4a^ and IL‐1β, two critical senescence‐related markers, were indirectly visualized by IHC staining images in all mice with needle‐induced IVDD (Figure 7H,I). Notably, the abnormal expression of these proteins was remarkably ameliorated by treatment with VN‐siSer compared to the VN‐siNC group (Figure 7H,I). This histological trend was consistent with the MRI findings. Importantly, although *Ace2* ^−/y^ mice showed more severe IVDD than WT mice, no significant differences in Pfirrmann and histological scores were observed between WT and *Ace2* ^−/y^ mice after inhibiting the expression of *Serpine1* using VN‐siSer (Figure 7D,G,I). This finding validates that nanovector‐mediated RNAi treatment has great potential as a general treatment for senescence‐associated IVDD.

Finally, the in vivo biocompatibility of VNs was evaluated at 1, 2, and 3 weeks after local injection into mouse NP tissue in terms of body weight, routine blood work, blood biochemistry indexes, and H&E staining of major organs (heart, liver, spleen, lung, and kidney) were performed (Figure S25A, Supporting Information). The overall morphology of the major organs showed no significant alternations upon the injection of VNs (Figure S25B, Supporting Information). In addition, the body weights of mice in the different groups were not significantly different (Figure S25C, Supporting Information). The results of routine blood tests demonstrated negligible variance among all the experimental groups (Figure S25D, Supporting Information). H&E staining of tissues also revealed no obvious structural changes between the control and treated groups (Figure S25E, Supporting Information). Blood biochemistry indices of VN‐injected mice were similar to those of untreated healthy mice, indicating the absence of organ injury caused by VNs (Figure S25F–H, Supporting Information). Taken together, these results suggest that VN‐siSer is a highly promising vector for gene delivery, featuring high transfection efficiency and favorable biocompatibility.

## 3 Discussion

Aging is the primary cause of many chronic degenerative diseases, including IVDD.^[^ ^34^ ^]^ Within this context, cell senescence is a distinctive cellular state provoked by diverse stressors and characterized by irreversible cessation of the cell cycle and secretion of SASP factors.^[^ ^9^ ^]^ The SASP encompasses an array of pro‐inflammatory cytokines, chemokines, and ECM‐degrading proteins, thereby amplifying local senescence through a feed‐forward loop.^[^ ^35^ ^]^ In recent years, there has been a burgeoning exploration of the intricate relationship between cellular senescence and IVDD.^[^ ^12^, ^17^, ^34^ ^]^ However, the underlying mechanisms and downstream signaling molecules remain largely unexplored. Here, we rigorously documented the pivotal role of ACE2 deficiency in NPC senescence and IVDD progression using sequencing analyses and experimental models. Remarkable downregulation of ACE2 in NP tissues was observed in both senescent NPCs and NP tissues from patients with severe IVDD, highlighting a strong correlation between the expression of ACE2 and the degree of disc degeneration.

**Figure 8:**
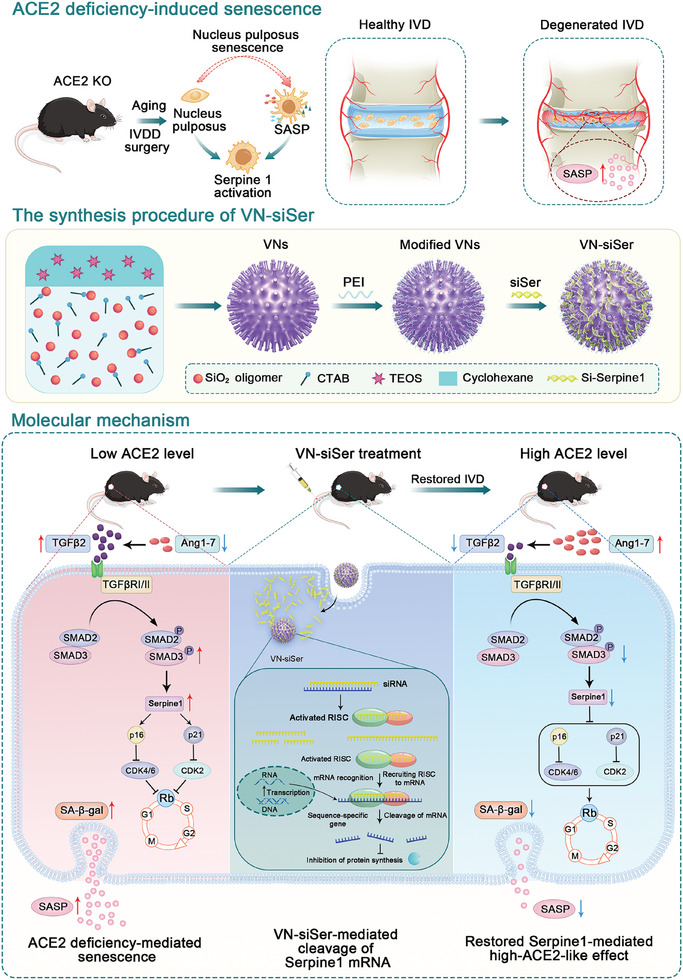
Schematic illustration of the mechanism of ACE2 regulation of NPC senescence and IVDD, and the use of VNs for enhanced gene therapy of IVDD. 1) NPCs undergo senescence during IVDD. 2) The process of making VN‐siSer included the formation and growth of mesoporous silica nanoparticles, the formation of the nucleation sites, the orientated growth of the silica nanotubes, and the loading of siSer onto VNs. 3) ACE2 expression was negatively correlated with IVDD severity. ACE2 deficiency increased the susceptibility of NPCs to senescence and aggravated IVDD. Nevertheless, ACE2 overexpression contracted aging and alleviated NPC senescence and IVDD. Further investigations revealed the mechanisms by which deficiency in ACE2/Ang‐(1–7)‐mediated activation of TGFβ2/Smads/Serpine1 contributes to NPC senescence and IVDD. Strategies that preferentially inhibit *Serpine1* in NPCs using a VN delivery system may represent novel and effective approaches to treat IVDD. NPC: nucleus pulposus cell; IVDD: intervertebral disc degeneration; ACE2: angiotensin‐converting enzyme 2; Serpine1: Serpin family E member 1.

In addition, loss‐ and gain‐of‐function mouse models were established to further substantiate the role of ACE2 in IVDD. Our observations demonstrated that ACE2 deficiency increases the susceptibility of NPCs to senescence and aggravates injury‐induced NPC dysfunction and IVDD, whereas ACE2 overexpression is protective against these pathological conditions. Integrated analysis of scRNA‐seq and bulk RNA‐seq revealed a new NPC‐Serpine1 subcluster in ACE2‐deficient aging mice. The findings from PPI analysis and in vitro experiments also mechanistically reveal that ACE2 deficiency can lead to the activation of the TGFβ2/Smad2/3 signaling pathway by promoting the transcription of *Serpine1*, ultimately triggering NPC senescence. The protective effect of ACE2 on NPCs and IVDD is mainly mediated by the Ang‐(1–7)/MasR axis. More importantly, we developed an engineered nanovector with a virus‐like surface topology for loading siRNA against Serpine1, a critical downstream molecule of ACE2. Systematic evaluations corroborated that genetically inhibiting the expression of *Serpine1* using this nanomedicinal delivery system can effectively rescue NPC senescence and IVDD with satisfactory outcomes (**Figure** **8**).

The classical role of the RAS is to regulate blood pressure and maintain water and electrolyte balance.^[^ ^36^ ^]^ Our previous study identified the overexpression of the ACE/angiotensin II (Ang II) axis within IVD tissue, linking Ang II to NPC senescence and the inflammatory response.^[^ ^16^ ^]^ ACE2 is the only known homolog of ACE with enzymatic activity and co‐localizes with ACE, exerting a counter‐regulatory role in balancing the ACE/Ang II axis.^[^ ^13^ ^]^ Recent studies have revealed the involvement of ACE2 in the regulation of orthopedic diseases. Hatem et al. reported the protective effects of ACE2/Ang‐(1–7) in estrogen‐deficient osteoporotic rats.^[^ ^37^ ^]^ More recently, Yasser et al. demonstrated that increasing the content of Ang‐(1–7) through ACE2 activation ameliorated functional, radiological, and histopathological alterations in a knee osteoarthritis rodent model.^[^ ^38^ ^]^ Nevertheless, to the best of our knowledge, the pathological role of the ACE2/Ang‐(1–7) axis in NPC senescence and IVDD progression has yet to be elucidated. To fill this gap, our study aimed to comprehensively investigate the underlying mechanisms and downstream signaling targets by employing both *Ace2* KO (*Ace2* ^−/y^) and overexpressing (h*Ace2* ^OE^) mice. The findings revealed mechanistic connections between ACE2 expression and NPC senescence, as well as NP tissue degeneration, which suggests that ACE2 plays a critical role in maintaining IVD homeostasis and that ACE2 dysfunction heightens the incidence of NPC senescence and IVDD.

To further explore the mechanism by which ACE2 regulates NPC senescence, we performed a bulk RNA‐seq analysis of *ACE2* knocking‐down human NPCs. The integration of scRNA‐seq and bulk RNA‐seq datasets revealed that ACE2 deficiency could upregulate the expression of *Serpine1*, a key driver of cellular senescence.^[^ ^31^ ^]^ Notably, the significant attenuation of the pro‐senescent effects observed upon silencing SERPINE1 underscores its role as a pivotal downstream mediator of ACE2. Mechanistically, ACE2 deficiency triggered the activation of TGFβ2/Smad2/3 signaling pathway, leading to increased expression of Serpine1 and the augmentation of the NPC‐Serpine1 cluster. This observation is consistent with a previous study by McCann et al., which demonstrated that TGFβ upregulated the expression of Serpine1 by inhibiting the miR‐30c.^[^ ^39^ ^]^ While a previous study indicated the essential role of TGFβ signaling in the growth of IVD,^[^ ^40^ ^]^ data presented in this study shows that the expression of TGFβ2 increased with the degree of degeneration. Moreover, specifically inhibiting TGFβ2 notably decreased the expression of Serpine1 in *ACE2*‐deficient NPCs. The results above may suggest that ACE2‐mediated activation of the TGFβ signaling pathway may indeed promote senescence. Interestingly, recent studies have suggested that activation of TGFβ signaling was detrimental to IVDD, whereas inhibition of aberrant TGFβ signaling could delay IVDD progression.^[^ ^41^ ^]^ Similarly, the protect effects of interfering with the TGFβ signaling pathway through activation of ACE2 were also validated in the treatment of osteoarthritis, a very similar degeneration‐related disease to IVDD.^[^ ^38^ ^]^ Therefore, evidence from our study illuminates the critical role of appropriate activation of the TGFβ2/SMAD2/3 pathway for preserving NPC homeostasis and mitigating IVDD progression.

Gene therapy has been explored for the treatment of various diseases since 1999,^[^ ^42^ ^]^ but its application to IVDD faces obstacles owing to the absence of suitable molecular targets. Based on these results, we identified *Serpine1* as a promising downstream signaling molecular target for intervention in ACE2 deficiency‐induced NPC senescence and IVDD. Additionally, screening for biocompatible nanovectors with optimal delivery and transfection efficiency is crucial for the effective treatment of IVDD. Lentiviruses and Adeno‐associated virus (AAV) are conventional viral vectors that deliver siRNAs in gene therapy.^[^ ^42^ ^]^ However, their application is severely impeded by potential immunogenicity, toxicity, and insertional mutagenesis.^[^ ^43^ ^]^ To overcome these limitations, researchers have focused on various nanocarriers, including polymer micelles, iron oxide, and Au nanoparticles, which have been used for siRNA delivery. Despite the great potential of nanomedicinal gene therapy, improvements are still required to enhance cellular uptake and transfection efficiency.^[^ ^44^ ^]^ Inspired by the unique surface structure of viruses, we developed and employed MSNs with a virus‐like surface topology as unique nanovectors to deliver the siRNA of *Serpine1*.^[^ ^29^ ^]^ Notably, the primary internalization pathways of VNs are caveolae‐mediated endocytosis and micropinocytosis. These pathways offer potential advantages by circumventing the negative effects of acidic lysosomal degradation on siRNA treatment efficacy.^[^ ^45^ ^]^ Benefiting from this nanotopology‐enhanced endocytosis, siRNA‐compacted VNs showed superior transfection efficiency compared to the commercial transfection agent Lip3000. In vitro and in vivo experimental data further demonstrated that inhibiting the expression of *Serpine1* by VN‐siSer not only effectively reversed ACE2‐deficiency‐induced NPC senescence and IVDD in aging mice, but also unexpectedly exhibited significant protective effects against oxidative stress‐triggered NPC senescence and injury‐mediated IVDD in normal mice. These encouraging treatment outcomes of VN‐siSer in NPC senescence and IVDD highlight the considerable potential of employing virus‐like nanovectors integrated with siRNA as an efficient RNAi strategy for IVDD treatment.

Finally, this study had several limitations. IVDD is a multifactor‐related degenerative disease. The experimental results revealed that ACE2 regulated NPC senescence and IVDD via TGFβ2/Smads‐mediated Serpine1 expression. However, ACE2‐related IVDD may only represent a subset of all IVDD cases. In addition, VN‐siSer was administered to the IVD via local injection, which may simultaneously damage the integrity of the IVD. Moreover, maintaining a consistent injection site for repeated treatments is challenging.

## 4 Conclusions

In conclusion, we identified the pivotal link between ACE2 expression and IVDD severity, shedding light on the detrimental effects of ACE2 deficiency on NPC senescence and IVDD progression. Specifically, evaluation of loss‐ and gain‐of‐function mouse models demonstrated that ACE deficiency markedly upregulated the level of SASP factors in NPCs and aggravated both injury‐ and ROS‐induced degeneration of NP tissues. Systematic in vitro and in vivo investigations also elucidate a rigorous mechanistic understanding that ACE2 deficiency leads to the activation of the TGFβ2/Smads signaling pathway and subsequent transcriptional upregulation of *SERPINE1*, which eventually accelerates NPC senescence and IVDD. Additionally, we developed an engineered Vns for the RNAi treatment of IVDD, targeting Serpine1. Taking advantage of the nanotopology‐enhanced transfection efficiency, VN‐siSer effectively rescued the senescence of NP tissues and suppressed the progression of IVDD in a range of mouse models. Overall, the findings of our study expand our knowledge of the mechanisms underlying ACE2 deficiency‐mediated activation of TGFβ2/Smads/ SERPINE*1* in NPC senescence and IVDD. Thus, strategies focusing on the preferential inhibition SERPINE1 in NPCs may represent promising and effective approaches for the treatment of IVDD.

## 5 Experimental Section

### Bulk RNA Microarray and Sequencing Data Analysis

The IVDD microarray datasets GSE15227^[^ ^46^ ^]^ and GSE70362^[^ ^47^ ^]^ were obtained from the Gene Expression Omnibus (GEO) database (https://www.ncbi.nlm.nih.gov/geo) to investigate ACE2 expression and IVDD. Dataset GSE245147^[^ ^48^ ^]^ was acquired to explore the relationship between cellular senescence and IVDD. Differential analysis of IVDD datasets was conducted using the limma package, selecting genes for further study as differentially expressed genes (DEGs) based on |logFC| > 0.2 and adjusted *p* value < 0.05. Venn diagrams were used to identify genes that were consistently upregulated or downregulated across the datasets. Notably, the bulk RNA‐seq dataset utilized in the study was normalized using the RPKM (reads per kilobase of transcript per million mapped reads) method. This choice ensured consistent gene expression quantification across samples and minimized biases from gene length and sequencing depth, enabling accurate comparisons that were crucial for interpreting senescence‐related gene activation and IVDD progression.

### Single‐cell Transcriptome Sequencing (scRNA‐seq) and Analysis

First, the intervertebral disc tissues (IVDs) were meticulously collected from *Ace2* knockout (*Ace2* ^−/y^) mice and submerged in ice‐cold phosphate‐buffered saline (PBS) to preserve tissue integrity during transportation to the laboratory for subsequent processing. Then, the IVD tissues were subjected to enzymatic digestion to prepare a single‐cell suspension crucial for 10X Genomics sequencing. The tissues were finely minced using sterile scissors and incubated in a digestion solution at 37 °C with gentle agitation for 2 h. The suspension was then filtered through a 70‐µm cell strainer to eliminate undigested tissue, and cell viability and cell counts were determined using a trypan blue exclusion assay. Subsequently, scRNA‐seq employed a Chromium Single Cell 3′ Library & Gel Bead Kit v3 (10X Genomics), following manufacturer instructions. An appropriate volume of the single‐cell suspension was loaded onto a chromium single‐cell controller to capture 10 000 cells per sample. After cell capture and barcoding, cDNA was synthesized and amplified. The quality and quantity of the amplified cDNA were assessed before library construction. Libraries were prepared according to the 10X Genomics protocol, including fragmentation, end repair, A‐tailing, adaptor ligation, and PCR amplification. Libraries were quantified using Qubit, and the quality was assessed using the Agilent 2100 Bioanalyzer. Sequencing was conducted using an Illumina NovaSeq 6000 system, targeting ≈50 000 reads per cell.

Finally, sequencing data were processed using the 10X Genomics Cell Ranger software suite for quality control, read alignment, and quantification of gene expression. Seurat v3 was used for preprocessing, quality control, normalization, and dimensionality reduction clustering.^[^ ^49^ ^]^ Genes were filtered with minimal expression in the three cells, excluded cells with fewer than 200 expressed genes per cell, and removed cells with a mitochondrial gene proportion exceeding 10%. Data normalization, identification of variable genes, and dimensionality reduction clustering were performed using the default parameters and standard workflows of the Seurat package. The Harmony package was employed to integrate the data across different samples.^[^ ^50^ ^]^ Single‐cell clusters were annotated using known marker genes for different cell types (https://panglaodb.se/index.html).

### Animals

Male *Ace2* knockout (*Ace2* ^−/y^) mice were procured from SPF Biotechnology Co., Ltd. (Beijing, China). The mice were maintained in a C57BL/6J background. Since female *Ace2* KO mice were not available, all experiments in this study were performed using male mice. The *Ace2* ^−/y^ mice were genotyped using genomic DNA extracted from tail biopsies, PCR amplification, and Sanger sequencing. *Ace2*‐overexpression mice (CAG‐h *ACE2* mice wherein the expression of *Ace2* was under the control of the CAG promoter) were purchased from SPF Biotechnology Co., Ltd (Beijing). All mice were maintained in plastic cages with free access to food and water and housed at 25 ± 2 °C with a 12 h light–dark cycle. All experiments followed the national ethical guidelines implemented by the Institutional Animal Care and Use Committee and were approved by local authorities.

### Synthesis of the Virus‐Like Nanovectors

VNs were synthesized by an epitaxial growth approach using a cationic surfactant as a template, tetraethyl orthosilicate (TEOS) as a silica source, NaOH as a catalyst, and an organic solvent such as cyclohexane as the oil phase. Typically, 1 g of hexadecyltrimethylammonium bromide (CTAB) and 0.8 mL of NaOH (0.1 m) were added to 50 mL of water and stirred gently at 60 °C for 2 h in a round bottom flask, then 20 mL of TEOS in cyclohexane (20% v/v) was added to the solution and kept at 60 °C with stirring for 48 h. The products were collected via centrifugation and washed several times with water and ethanol. Finally, the VNs were dispersed in 50 mL of acetone and refluxed at 50 °C for 12 h to remove CTAB templates. Then, the samples were washed with ethanol and dried under vacuum at 45 °C for 8 h. In order to facilitate electrostatic interactions between VNs and siRNA, positively charged polyethylenimine (PEI) was added to the solution containing VNs and stirred for 12 h. Then, the VN‐siSer NPs were prepared by mixing siRNA followed by a gentle vortex for 30 s. The solutions were left undisturbed for 30 min to enable the formation of stable VN‐siSer.

### siRNA for ACE2 and Serpine1 Transfection Assay I

Small interfering RNAs (siRNAs) used for ACE2 and Serpine1 were purchased from GenePharma (Shanghai, China). The NPCs were transplanted into 12‐well plates. After the cell density reached 60%–70%, Lipofectamine 3000 reagent (Invitrogen) was used to transfect the siRNA for ACE2 or Serpine1 into the NPCs, following the manufacturer's protocol. The sequence for *ACE2* was as follows: CCAGGUUUGAAUGAAAUAATT (sense), UUAUUUCAUUCAAACCUGGTT (anti‐sense); the sequence for human *Serpine1* was as follows: CAGACAGUUUCAGGCUGACUUCACG (sense), CGUGAAGUCAGCCUGHAAACUGUCUG (anti‐sense); the sequence for mouse *Serpine1* was as follows: CAGAAGUGGAAAGAGCCAGAUUUAU (sense), UUGACUUUGAAUCCCAUAGCAUCUU (anti‐sense) 31. After 24 h, RT‐qPCR was performed to verify the effect of gene silencing.

### RNA Reverse Transcription, and Quantitative Real‐Time Polymerase Chain Reaction (RT‐qPCR)

After the acquisition of total mRNA from NPCs, HiScript III RT SuperMix for qPCR Kit (R323‐01, Vazyme, Nanjing, China) was used to reverse transcribe the mRNA. Gene expression was quantified using SYBR qPCR Master Mix (Q711‐02, Vazyme) on a Real‐Time PCR system (Applied Biosystems, Foster City, USA). β‐actin was used as a reference gene. Relative gene expression was quantified using the formula: 2^−∆∆Ct^. The primers used in this study are listed in Table S2 (Supporting Information).

### Establishment of Mechano‐ and Puncture‐Induced Mouse IVDD Models

All animal experiments conformed to the National Institutes of Health Guide for the Care and Use of Laboratory Animals. This method was described in detail in the previous study. In the present study, two mouse models were used: a puncture‐induced IVDD model and a lumbar instability (LIS)‐induced IVDD model^.[^ ^51^, ^52^ ^]^ These two models were commonly used to study the mechanisms and therapeutic effects of IVDD. Needle‐induced IVDD was used to mimic injury‐related IVDD, similar to that observed in patients with AF rupture. LIS‐induced IVDD was used to mimic mechanical stress‐related IVDD, similar to lumbar instability.

To establish a puncture‐induced IVDD mouse model, the mouse tail disc at Co 4/5 was punctured using a sterilized needle (2 mL injection syringe). Next, the needle was rotated 180° and kept in place for 30s. The LIS model was used to establish mechano‐induced IVDD. Briefly, mice were anesthetized with isoflurane, and the 3rd–5th lumbar (L3–L5) spinous processes, along with the supraspinous and interspinous ligaments, were resected to induce instability of the lumbar spine. In the sham group, only the paraspinal‐dorsal muscles were isolated.

All the mice were housed in an animal room with a constant temperature of 20–22 °C and humidity of 50%–65% under a 12 h light/dark cycle, and were given free access to food and water. The mice were sacrificed four weeks postoperatively and the IVD tissue was harvested for subsequent MRI and histochemical experiments.

### Radiographic Analysis

T2‐weighted MRI of the IVD was performed using a 3.0T MRI (United Imaging, China) to evaluate the signal and structural changes in the IVD of the mice. A higher signal indicated a healthier IVD. The Pfirrmann grade of IVDD in different groups of mice was evaluated using MRI T2W1 images. The MRI grading system was divided into five levels ranging from Grade I (normal) to Grade V (severe degeneration).

### Histological Staining and Analysis

Human and mouse IVD tissues were performed using similar procedures. Briefly, after IVD tissue was acquired, 4% paraformaldehyde was used to fix the tissue for 1 d, followed by decalcification for 7 d. The tissues were embedded in paraffin, and the paraffin sections were deparaffinized in graded xylene, rehydrated in graded alcohol solutions, and washed with PBS. Next, the sections were stained with H&E or SOFG using appropriate kits (G1076, GP1051, Servicebio, Wuhan, China). A modified histological grading system was used to quantify the histological IVD scores (Table S3, Supporting Information). For immunohistochemical staining, the sections were sequentially blocked with 0.1% Triton X‐100 and 5% bovine serum albumin (BSA) solution, followed by incubation with the primary antibody overnight. Primary antibodies including p16^INK4a^ (R23897/380963, Zenbio), p21 (YT3497, Immunoway), COL2A1 (GB11021, Servicebio), Serpine1 (AF03419, AiFang Biological), and IL‐1β (AF5103, Affinity). A secondary antibody (GB23303; Servicebio) was used to bind to the primary antibody. Images were captured using a light microscope (Olympus). For immunofluorescence analysis, histological sections or NPCs were permeabilized using 0.1% Triton X‐100 for 10–15 min, followed by blocking with a 5% BSA solution for 25–30 min. The samples were incubated overnight with primary antibodies. Primary antibodies included MMP3(340612, Zenbio), ACAN (GB11373, Servicebio, or ab186414, Abcam), p53 (YM3052, Immunoway), p16^INK4a^ (R23896/ R23895, Zenbio), p21 (YT3497, Immunoway), IL‐1β (AF5103, Affinity), γH2AX (GB111841, Servicebio), PCNA (GB12010, Servicebio), and Ki67 (GB121141, Servicebio), p‐Smad2 (AF3449, Affinity), p‐Smad3 (AF3362, Affinity), ACE2 (AF300783, AiFang Biological), and Serpine1 (AF03419, AiFang Biological). On the second day, the samples were incubated with Alexa Fluor 488‐ and FITC‐conjugated secondary antibodies (GB22303, GB21301, Servicebio) for 1 h. The nuclei were staining with 4′,6‐diamidino‐2‐phenylindole solution (G1012‐100ML, Servicebio). Finally, sections or NPCs were washed, air‐dried, and treated with antifluorescence‐quenching tablets. Fluorescence was detected using a fluorescence microscope (Olympus). Sections were stained using a Sirius Red Staining kit (GP1138, Servicebio).

### Statistical Analysis

The data in this study were obtained through independent experiments or repeated measurements, with sample sizes of *n =* 3, 4, or 5. Data were presented as mean ± standard deviation (SD). Two‐tailed unpaired Student's *t*‐test and two‐way analysis of variance (ANOVA) followed by Tukey's post‐hoc test were used to determine significant differences between groups. For ordinal data, significant differences were assessed using the Kruskal–Wallis test followed by Dunn's post hoc test. Statistical analyses were performed using GraphPad Prism 9 software (GraphPad Software Inc., La Jolla, CA, USA). The results were considered statistically significant if the *p*‐value was <0.05.

### Ethics Statement

The human IVD tissues used in this study were obtained intraoperatively from the authors’ hospital. Ethical approval for the use of human tissue was granted by the Institutional Human Ethics Review Board of the Naval Medical Center (approval no. 2023030310). Permission to use animal models was obtained from the Naval Medical Center (approval no. NMC‐2023018).

## Conflict of Interest

The authors declare no conflict of interest.

## Supporting information



Supporting Information

## Data Availability

The data that support the findings of this study are available from the corresponding author upon reasonable request.
