# Supplementary material for: Rescuing ACE2‐Deficiency‐Mediated Nucleus Pulposus Senescence and Intervertebral Disc Degeneration by a Nanotopology‐Enhanced RNAi System
Source: Adv Sci (Weinh). 2025 Jan 13;12(9):2412908. doi: 10.1002/advs.202412908 (PMC11884558; doi:10.1002/advs.202412908)
Supplement: Supplementary file 1 — Supporting Information [file ADVS-12-2412908-s001.docx]

Supporting Information

Rescuing ACE2-deficiency-mediated nucleus pulposus senescence and intervertebral disc degeneration by a nanotopology-enhanced RNAi system

*Kaiqiang Sun^†^, Zijian Kang^†^, Chen Yan^†^, Yan Yu^†^, Linhui Han^†^, Yangyang Shi, Liang Chen*********, Jiangang Shi* ********, Yu Chen*********, Jingchuan Sun********

† These authors contributed equally to this work.

*Corresponding Author: Jingchuan Sun, Yu Chen, Jiangang Shi, Liang Chen

**This file includes:**

Experimental sections

Figs. S1 to S25

Tables S1 and S3

**Experimental sections**

*Cell cycle analysis*: We assessed the cell cycle phases using the Seurat package's cell cycle scoring method.^[51]^ Specifically, we assigned cells to the G1, S, or G2M phases based on canonical markers and calculated cell cycle scores using the cell cycle scoring function. This method allows for the quantification and regression of cell-cycle effects during data preprocessing, thereby enhancing the accuracy of downstream analyses.

*Pseudo-time analysis of NPC clusters*: Monocle 2 package was used for pseudo-time analysis of the cell timing sequence in each sample.^[52]^ scRNA-seq data were processed by normalization, filtering of low-quality cells, and selection of highly variable genes. For clustering, differential expression analysis was performed on genes expressed in more than 10 cells. The DDRTree method was used to identify pseudo-time-dependent gene expression across all cells. Heatmaps were used to visualize the expression of all identified pseudo-time-dependent genes. KEGG analysis was performed using ClusterProfiler.

*Transcription factor activity analysis:* Single-cell transcription factor activity was analyzed using the SCENIC software. The analysis count matrix was obtained from single-cell RNA-seq data. Co-expression modules were then inferred using GRNBoost2 or GENIE3 algorithms within SCENIC. Following this, cisTarget databases were employed to identify regulons, which are gene sets co-regulated by shared transcription factors. The activity of these regulons was subsequently scored across individual cells using the AUCell algorithm. Finally, the heatmap and dot plots were utilized for the exploration and visualization of the results.

*GO and KEGG enrichment analysis*: The R package clusterProfiler conducted GO and KEGG annotation analysis on common differential genes, with entry selection criteria of p value < 0.05 and FDR value (q value) < 0.05 deemed statistically significant. Cnetplot and bubble charts showcased the enrichment results. The String database analyzed protein interactions, and Cytoscape software created interaction network diagrams.

*Gene set enrichment analysis (GSEA)*: Initially, we used Findmarkers function to calculated differential genes between the NPC-Serpine1 subgroup compared to other subgroups, sorted by logFC values. Then, the R package clusterProfiler conducted enrichment analysis on all genes within the consolidated GEO dataset, with GSEA parameters including 1000 permutations, gene set size limits of 10 to 500 genes, and p value adjustment using the Benjamini-Hochberg.

*The acquisition of human NP tissue samples*: Slightly, mildly, and severely degenerated NP tissue, based on grades II, III, and IV/V, respectively, determined by a Pfirrmann score, were obtained from patients who underwent disc replacement and fusion due to spine fracture, spinal stenosis, or disc herniation in our hospital. The basic patient information is provided in **Table S1**.

*Bulk transcriptome sequencing of human: NPCs and bioinformatics analysis*: Analysis of mRNA-seq data of human primary NPCs were performed in the laboratory with the help of Genekinder Medicaltech (Genekinder Medicaltech Co., Ltd., Shanghai, China). The R package Deseq2 was used to analyze the DEGs.^[53]^ Subsequent additional analysis was performed on differentially expressed mRNAs, which showed a log2(FC) value > 1.5 and a false discovery rate < 0.05. An illustration of the DEGs between the two samples is shown in volcano and heatmap plots using the ggplot2 and heatmap packages.

*Isolation and culture of primary NPCs:* For human primary NPCs, after collecting the human IVD tissue intraoperatively, the tissue was kept in sterile saline solution and transported to the laboratory immediately. Then, the AF tissue was removed to obtain fresh gel-like NP tissues, followed by successive digestion using trypsin (0.25% EDTA+) (25200072, Gbico, ThermoFisher Scientific) for 30 minutes and collagenase type II (0.2%) (A004174-0100, Sangon Biotech) for another 60 minutes in a water bath of 37 °C. However, for mouse IVD tissue, the digestion was simplified. After acquired the NP tissue with the assistance of a dissecting microscope, the tissue was digested using only collagenase type P (0.0125%, Roche Diagnostics, 11213865001) for 60 minutes. Subsequently, the digested tissue was passed through a cell strainer (CSS013070, Qingning Bio.) with a pore size of 70 μm. Finally, the NPCs were cultured in a 37 °C incubator using a DF-12 culture medium (11330057, Gbico, ThermoFisher Scientific) containing 15% FBS (10099-141C, Gbico) and 1% penicillin-streptomycin (15140163, Gbico, ThermoFisher Scientific).

*Senescence β-galactosidase staining*: Senescent NPCs were stained with Senescence β-Galactosidase Staining Kit (C0602, Beyotime, Shanghai, China), according to the manufacturer's instructions. After the NPCs in the 6-well plates were treated, the cells were washed twice with PBS. Then, SA-β-Gal fixation solution (1 mL) was added to each well and fixed at room temperature for 10 min. Subsequently, the cell fixation solution was removed and washed three times with PBS, followed by the addition of the staining working solution overnight at 37 °C. Images were captured using a high-resolution microscope (NIKON Eclipse Ti, Tokyo, Japan), and the blue cells were senescent NPCs.

*Analysis of mitochondrial membrane potential (Δ𝜓M):* JC-1 staining kit was used to measure the mitochondrial membrane potential (C2001S, Beyotime Biotechnology, Inc., Shanghai, China) according to the instructions. NPCs were stained with JC-1 and captured using a fluorescence microscope (Olympus, Japan).

*Detection of intracellular ROS:* Intracellular ROS was measured using a ROS assay kit (S0033S, Beyotime, Shanghai, China) according to the instructions. ROS was visualized with a DCFH-DA probe and imaged under a fluorescence microscope (Olympus, Japan).

*Cell viability assay:* As described in the instructions, NPCs were tested for viability using the cell counting kit-8 (CCK8) (No. C0005, TargetMol Chemicals Inc., USA). Briefly, after the NPCs were stimulated with VNs in a dose-dependent manner for 24 h, the cells were incubated with working solution (100 μL serum-free medium and 10 μL CCK8 solution) for 2 h at 37 ℃ (n=4/group). A THERMO automatic microplate reader (Thermo Fisher Scientific, USA) was used to measure absorbances at 450 nm.

*Live/dead staining assay of NPCs*: The prepared NPCS were stained with 500 μl of live/dead cell dye solution for 15 min and detected using an LSCM (G1707, Servicebio). The viable NPCs with esterase activity appeared in green fluorescence, while the dead cells were in red fluorescence.

*Endosome/lysosomal escape assay*: Human primary NPCs (4 × 10^4^) were seeded onto cellular slides in 6-well plates for 24 h to ensure cell adhesion. FAM-labelled VN-siRNAs were added to the culture medium and cocultured with NPCs for 12, 24, or 48 h. At the end of the experiment, the NPCs were washed to remove FAM-labelled VN-siRNAs, followed by visualization of lysosomes using LysoTracker® Red DND-99 for 30 min at 37 °C. Then, the culture medium was changed to fresh medium. The slices were sealed, and images were obtained using confocal scanning laser microscopy (ZEISS LSM880, Germany).

*Gel retardation assay*: To measure siRNA encapsulation efficiency in VNs, a gel retardation assay was performed. The complexes of nanoparticles and siRNA at various weight ratios of VNs to siRNA (1:1, 2:1, 5:1, 10:1, 20:1, and 40:1) were first prepared by gently vortexing the mixtures of 1 µL of siRNA solution (1.5g/L) and 10 µL of VNs solution with different concentrations in a tube. The mixtures were then incubated with siRNA for 20 min at room temperature to allow complex formation. Then, the complex solution was combined with 1X loading buffer and injected into the lanes for electrophoresis (15 µL/lane), with free siRNA as control. Electrophoresis was conducted on a 2% agarose gel containing 10 µl of 0.5 UA/mL ethidium bromide in 0.5X Tris/borate/EDTA buffer at 90 mV for 20 min. The resulting gel was imaged using a UV transilluminator (Bio-Rad, USA).

*Western blotting*: Super-RIPA lysis buffer with protease (P1005, Beyotime) and a phosphatase inhibitor cocktail (C0002, TOPSCIENCE) was used to lyse human NPCs or NP tissue. After quantification, the total NPC protein was loaded onto a sodium dodecyl sulfate-polyacrylamide gel (10%, PG112, Shanghai Epizyme Biomedical Technology Co., Ltd, China) and transferred onto polyvinylidene fluoride membranes (HVLP04700, Millipore, Sigma–Aldrich, USA). After blocking the membranes with 5% dry skim milk, they were incubated with primary antibodies, followed by incubation with secondary antibodies. Antibodies included p16^INK4a^ (R23897/380963, Zenbio), p21 (YT3497, Immunoway), IL-1β (AF5103, Affinity), MMP13 (AF5355, Affinity), SERPINE1 (AF03419, AiFang Biological), ACAN (GB11373, Servicebio, or DF7561, Affinity), COL2A1(ab34712, Abcam), MMP3(340612, Zenbio), p-Smad2 (AF3449, Affinity), Smad2 (AF6449, Affinity), p-Smad3 (AF3362, Affinity), Smad3 (AF6362, Affinity), and GAPDH (250133, Zenbio).

*Quantification of SASP-related cytokines*: The levels of SASP-related cytokines in the supernatant of NPCs from different groups were measured using the enzyme-linked immunosorbent assay (ELISA) kits, including, IL-1β (ED-10351, ED-20174, LunChangShuoBiotech, Ximen, China), IL-6 (ED-10377, ED-20188, LunChangShuoBiotech), IL-13 (ED-12599, ED-20167, LunChangShuoBiotech), MMP3 (ED-10753, ED-20379, LunChangShuoBiotech), and MMP13 (ED-14942, ED-20377, LunChangShuoBiotech).

*Radiographic analysis*: T2-weighted MRI of the IVD was performed using a 3.0T MRI (United Imaging, China) to evaluate the signal and structural changes in the IVD of the mice. A higher signal indicated a healthier IVD. The Pfirrmann grade of IVDD in different groups of mice was evaluated using MRI T2W1 images. The MRI grading system was divided into five levels ranging from Grade I (normal) to Grade V (severe degeneration).

*Histological staining and analysis*: human and mouse IVD tissues were performed using similar procedures. Briefly, after IVD tissue was acquired, 4% paraformaldehyde was used to fix the tissue for 1 d, followed by decalcification for 7 d. The tissues were embedded in paraffin, and the paraffin sections were deparaffinized in graded xylene, rehydrated in graded alcohol solutions, and washed with PBS. Next, the sections were stained with H&E or SOFG using appropriate kits (G1076, GP1051, Servicebio, Wuhan, China). A modified histological grading system was used to quantify the histological IVD scores (**Table S3**). For immunohistochemical staining, the sections were sequentially blocked with 0.1% Triton X-100 and 5% bovine serum albumin (BSA) solution, followed by incubation with the primary antibody overnight. Primary antibodies including p16^INK4a^ (R23897/380963, Zenbio), p21 (YT3497, Immunoway), COL2A1 (GB11021, Servicebio), Serpine1 (AF03419, AiFang Biological), and IL-1β (AF5103, Affinity). A secondary antibody (GB23303; Servicebio) was used to bind to the primary antibody. Images were captured using a light microscope (Olympus). For immunofluorescence analysis, histological sections or NPCs were permeabilized using 0.1% Triton X-100 for 10–15 min, followed by blocking with a 5% BSA solution for 25–30 min. The samples were incubated overnight with primary antibodies. Primary antibodies included MMP3(340612, Zenbio), ACAN (GB11373, Servicebio, or ab186414, Abcam), p53 (YM3052, Immunoway), p16^INK4a^ (R23896/ R23895, Zenbio), p21 (YT3497, Immunoway), IL-1β (AF5103, Affinity), γH2AX (GB111841, Servicebio), PCNA (GB12010, Servicebio), and Ki67 (GB121141, Servicebio), p-Smad2 (AF3449, Affinity), p-Smad3 (AF3362, Affinity), ACE2 (AF300783, AiFang Biological), and Serpine1 (AF03419, AiFang Biological). On the second day, the samples were incubated with Alexa Fluor 488- and FITC-conjugated secondary antibodies (GB22303, GB21301, Servicebio) for 1 h. The nuclei were staining with 4′,6-diamidino-2-phenylindole solution (G1012-100ML, Servicebio). Finally, sections or NPCs were washed, air-dried, and treated with antifluorescence-quenching tablets. Fluorescence was detected using a fluorescence microscope (Olympus). Sections were stained using a Sirius Red Staining kit (GP1138, Servicebio).

**Supplementary Figures and caption**


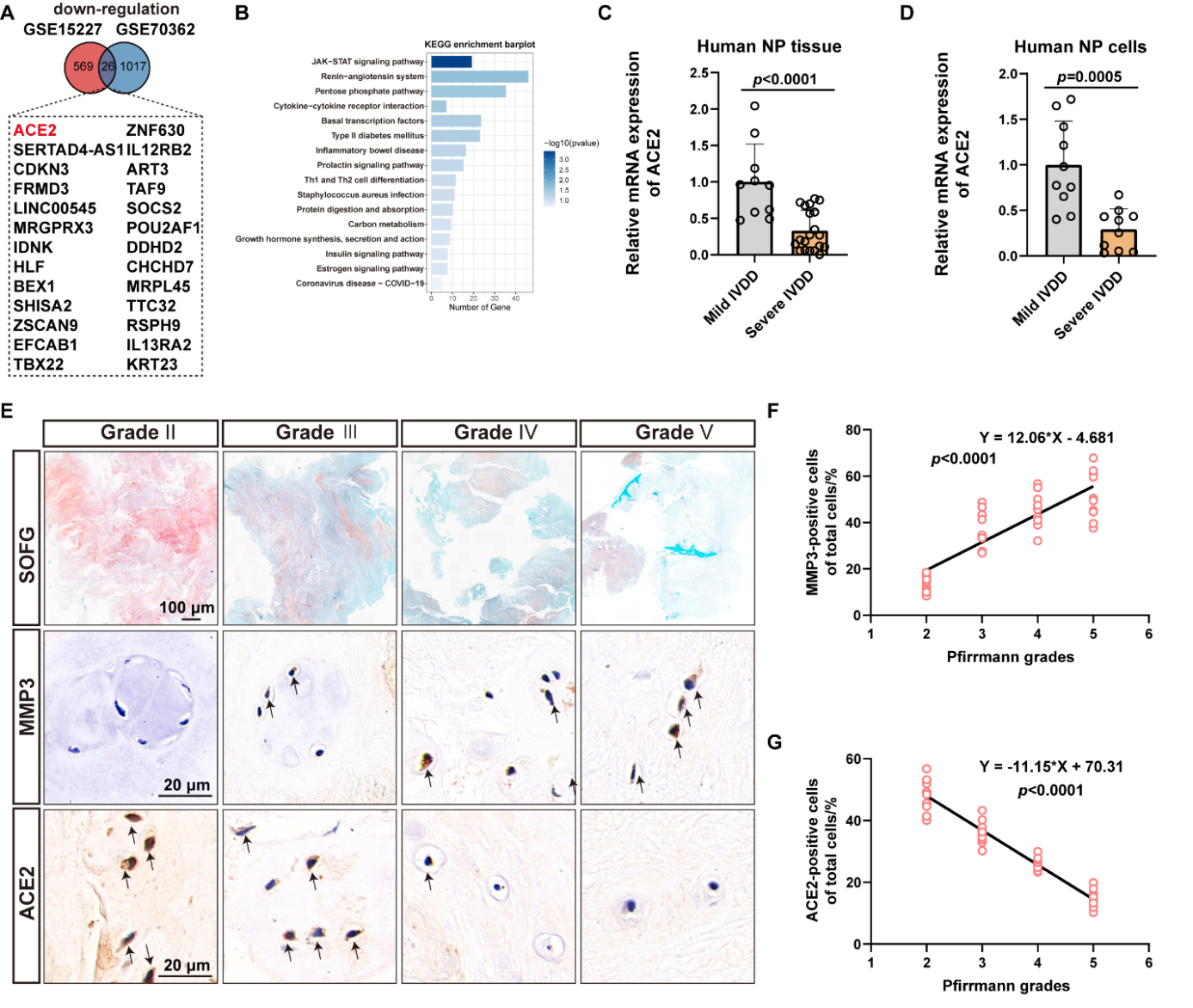


**Figure S1. ACE2 expression was negatively correlated with IVDD severity**. (A) An analysis of RNA sequencing data deposited in the Gene Expression Omnibus database (GSE15227 and GSE70362) showed that the expression of ACE2 was decreased in degenerated NP tissue. (B) The KEGG analysis of the differentially expressed genes from FigureS1. A. (C) The mRNA expression of ACE2 in NP tissue from mild IVDD and severe IVDD as shown by RT-qPCR (n=10 in mild IVDD group and n=20 in severe IVDD group). Two-tailed unpaired Student’s t test was used. (D) The mRNA expression of ACE2 in NPCs mild IVDD and severe IVDD as shown by RT-qPCR (n=10 per group). Two-tailed unpaired Student’s t test was used. (E) Representative images of SOFG staining and IHC for MMP3 and ACE2 in human NP tissue with different degeneration grades based on Pfirrmann score (Scale bar=100 μm, 20μm). (F and G) Single-factor linear regression analysis of the relationship between the percentage of MMP3- and ACE2-positive NPCs and Pfirrmann grades human NP samples. KEGG: Kyoto Encyclopedia of Genes and Genomes; NPC: Nucleus pulposus cell; SOFG: Safranin O and Fast green; MMP3: Matrix Metalloproteinase 3; ACE2: Angiotensin-converting enzyme 2. *P < 0.05, **P < 0.01, ***P < 0.001, and ****P < 0.0001.


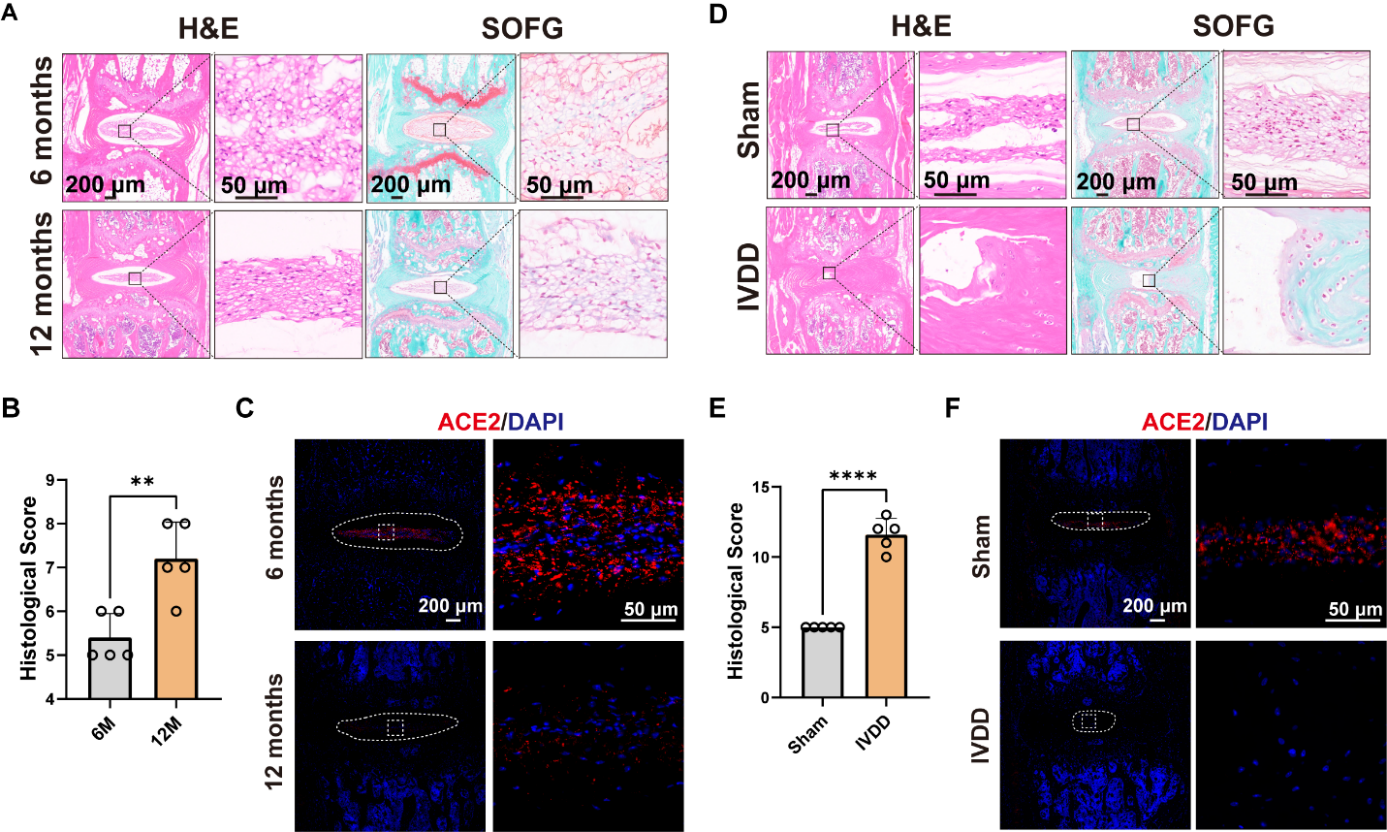


**Figure S2. Aging and degenerated NP tissue of mice were accompanied by decreased expression of ACE2.** (A) Representative images of H&E and SOFG staining of IVD from mice at the age of 6 and 12 months, respectively. (B) Histological score of IVD tissue from mice at the age of 6 and 12 months, respectively (n=5). Two-tailed unpaired Student’s t test was used. (C) Representative images of IF staining for ACE2 in IVD from mice at the age of 6 and 12 months, respectively. (D) Representative images of H&E and SOFG staining of IVD from the mice in sham and IVDD (needle injury) group, respectively. (E) Histological score of IVD tissue from mice in sham and IVDD (needle injury) group, respectively (n=5). Two-tailed unpaired Student’s t test was used. (F) Representative images of IF staining for ACE2 in IVD from mice in sham and IVDD (needle injury) group, respectively. H&E: Hematoxylin-eosin staining; SOFG: Safranin O and Fast green; IVD: Intervertebral disc; IF: Immunofluorescence; IVDD: Intervertebral disc degeneration; ACE2: Angiotensin-converting enzyme 2 *P < 0.05, **P < 0.01, ***P < 0.001, and ****P < 0.0001.


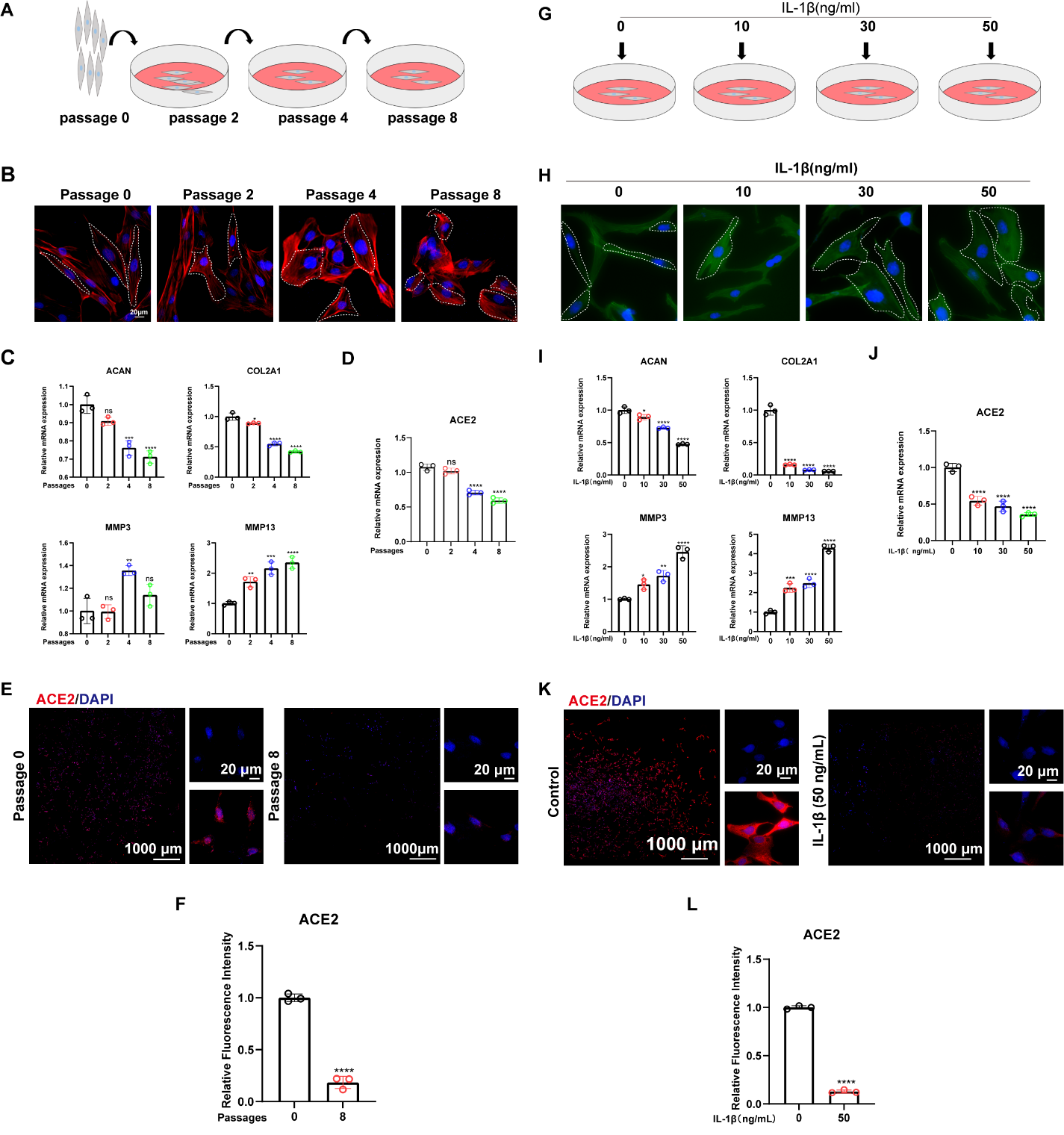


**Figure S3. The changes of ACE2 expression in aging and injured NPCs in vitro.** (A) The strategy for NPC passaging. (B) Representative images of phalloidin staining of NPCs at the passage from 0 to 8. (C) The mRNA expression of ACAN, COL2A1, MMP3, and MMP13 in NPCs at the passage from 0 to 8. Two-tailed unpaired Student’s t test was used. (D) The mRNA expression of ACE2 in NPCs at the passage from 0 to 8. Two-tailed unpaired Student’s t test was used. (E) Representative images of IF staining for ACE2 of NPCs at the passage from 0 to 8. (F) Quantitative results of IF staining for ACE2 of NPCs at the passage from 0 to 8. Two-tailed unpaired Student’s t test was used. (G) The strategy for IL-1β-induced NPC injury. (H) Representative images of phalloidin staining of NPCs treated by IL-1βwith different concentrations. (I) The mRNA expression of ACAN, COL2A1, MMP3, and MMP13 in NPCs treated by IL-1βwith different concentrations. Two-tailed unpaired Student’s t test was used. (J) The mRNA expression of ACE2 in NPCs treated by IL-1β with different concentrations. Two-tailed unpaired Student’s t test was used. (K) Representative images of IF staining for ACE2 of NPCs treated with or without IL-1β. (L) Quantitative results of IF staining for ACE2 of NPCs treated with or without IL-1β. Two-tailed unpaired Student’s t test was used. NPC: Nucleus pulposus; IF: Immunofluorescence. *P < 0.05, **P < 0.01, ***P < 0.001, and ****P < 0.0001.


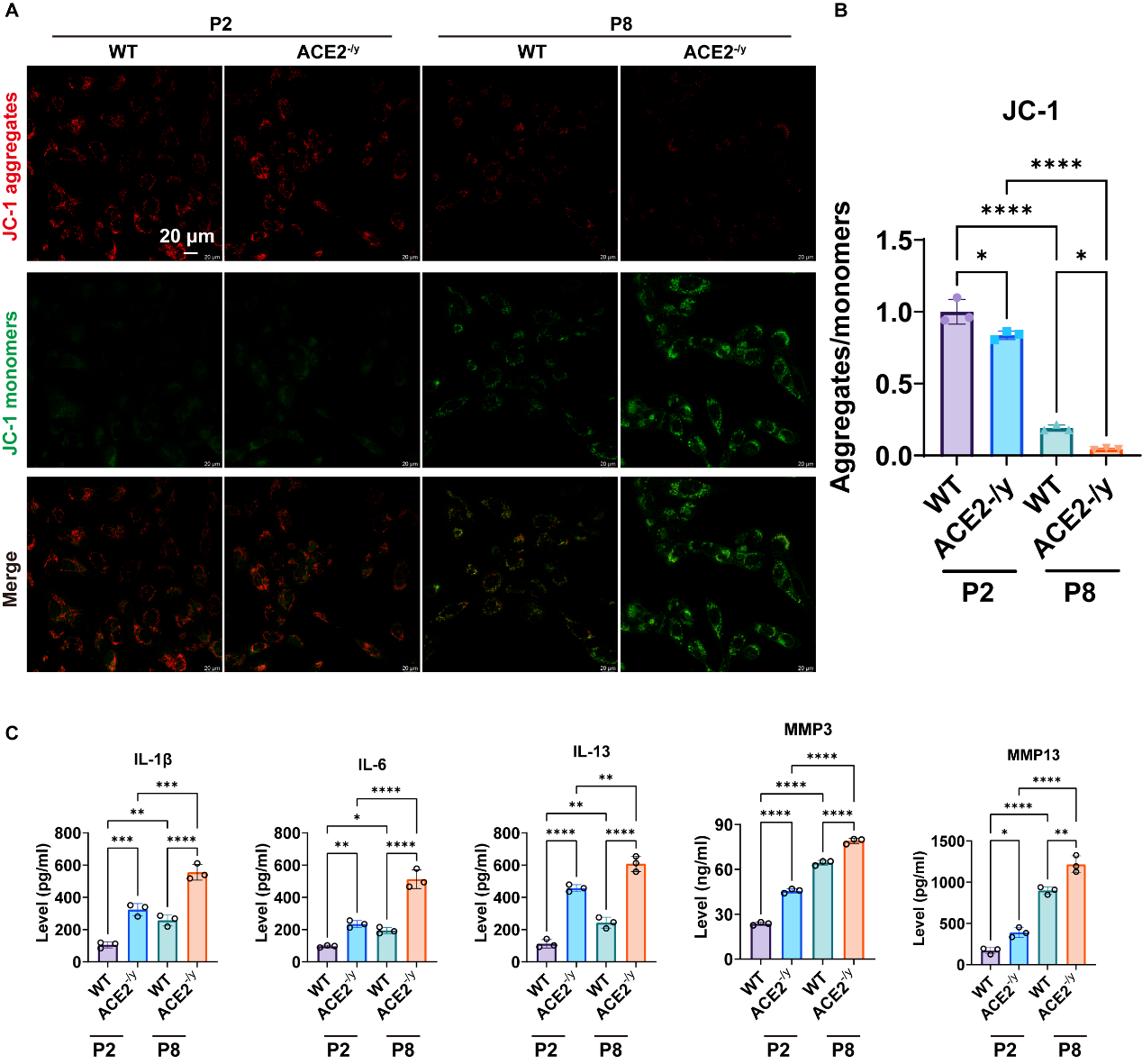


**Figure S4. ACE2 deficiency increased the susceptibility of NPCs to mitochondrial dysfunction and SASP**. (A) Mitochondrial membrane potential (Δ𝜓M) of passage 2 or 8 NPCs from Ace2-/y mouse and WT littermates at the age of 3 months. (Scale bar=20 μm) (n=3 per group). (B) Quantitative results of Δ𝜓M in different groups. (C) ELISA assay of SASP-related cytokines in the supernatant of NPCs from Ace2-/y mouse and WT littermates at the age of 3 months (n=3 per group). P values were determined by two-way ANOVA with Tukey's post hoc test. All data are presented as mean ± SD. ACE2: Angiotensin-converting enzyme 2; NPC: Nucleus pulposus cell; SASP: Senescence-associated secretory phenotype. *P < 0.05, **P < 0.01, ***P < 0.001, and ****P < 0.0001.


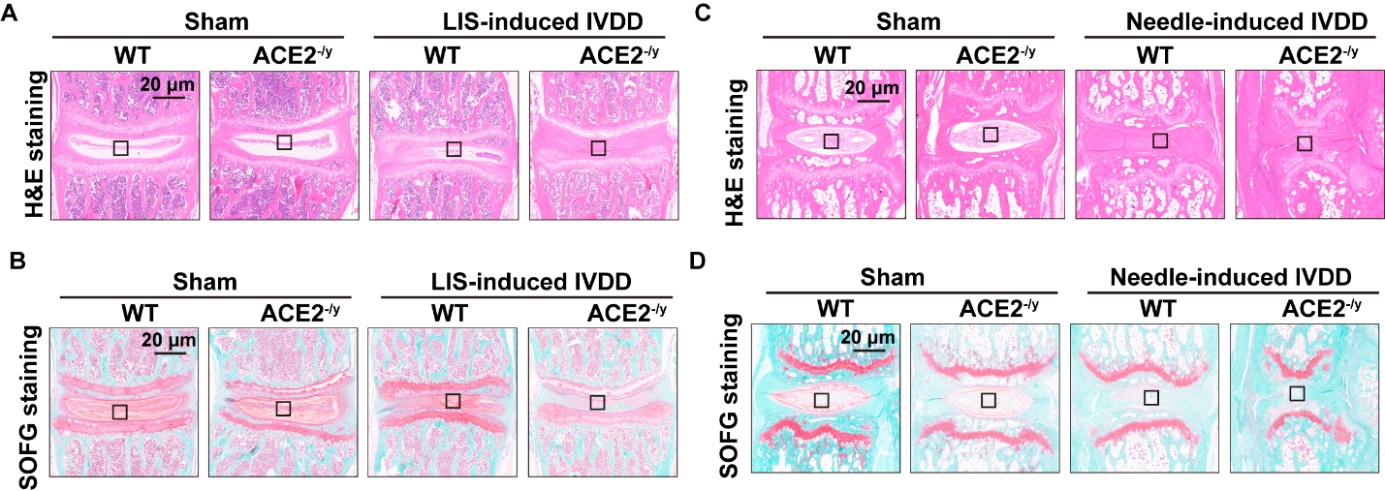


**Figure S5. Histological analysis of IVD from WT and *Ace2*^-/y^ mice with or without the surgery of lumbar instability or tail needling.** (A) Representative images of H&E staining of IVD tissue from WT and Ace2-/y mice with or without the surgery of lumbar instability (Scale bar=20 μm). (B) Representative images of SOFG staining of IVD tissue from WT and Ace2-/y mice with or without the surgery of lumbar instability (Scale bar=20 μm). (C) Representative images of H&E staining of IVD tissue from WT and Ace2-/y mice with or without the surgery of tail needling (Scale bar=20 μm). (D) Representative images of SOFG staining of IVD tissue from WT and Ace2-/y mice with or without the surgery of tail needling (Scale bar=20 μm). H&E: Hematoxylin and eosin; SOFG: Safranin-O fast Green; IVD: Intervertebral disc.


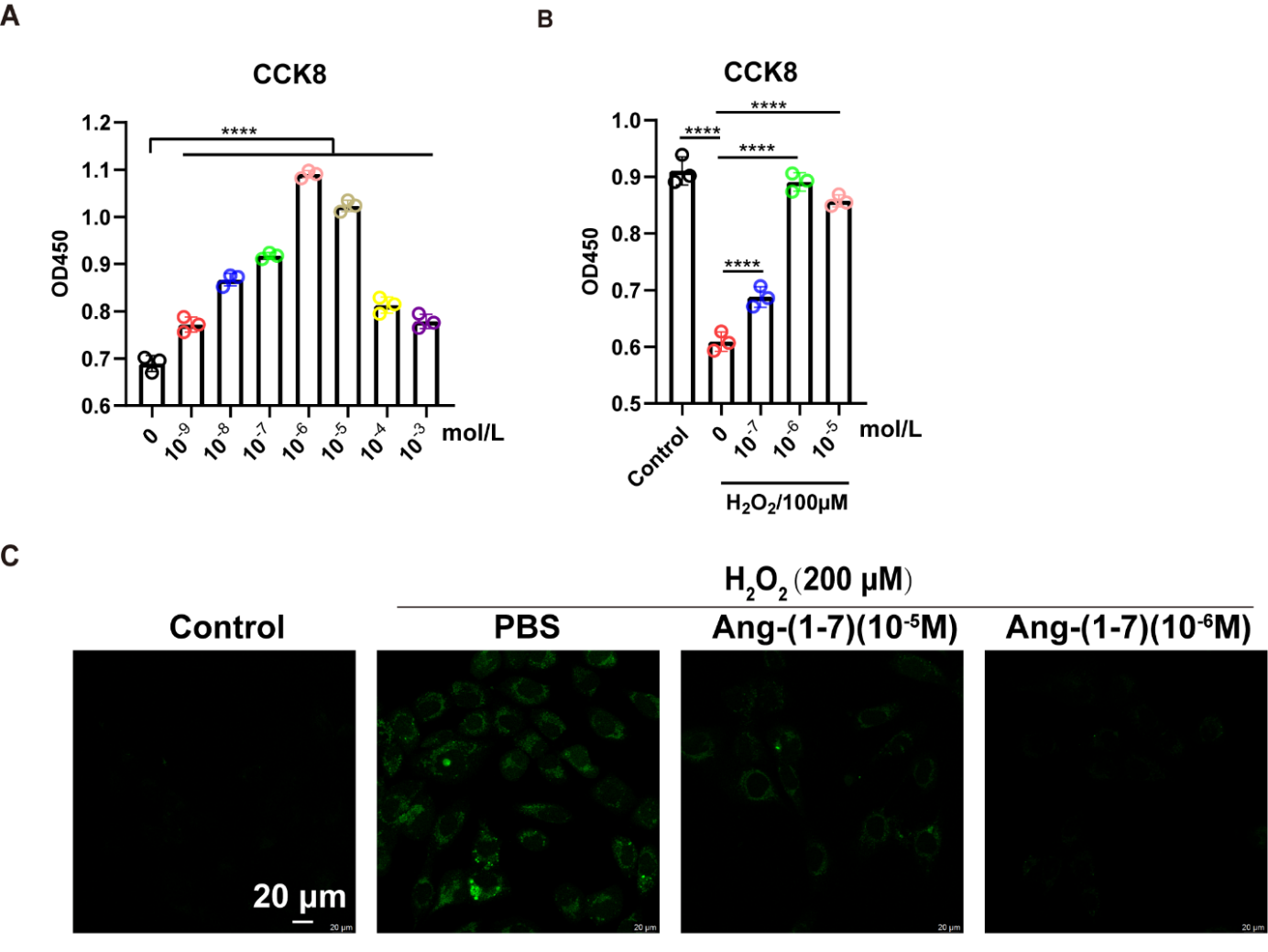


**Figure S6. Ang-(1-7) showed protective effects on NPCs.** (A) Cell viability assay of NPCs treated with Ang-(1-7) of different concentrations (n=3 per group). Two-tailed unpaired Student’s t test was used. (B) Cell viability assay of NPCs treated by H2O2 with or without Ang-(1-7) (10-7-10-5 M) (n=3 per group). P values were determined by two-way ANOVA with Tukey's post hoc test. (C) DCFH-DA staining of NPCs treated by H2O2 with or without Ang-(1-7) (10-6 and 10-5 M) (Scale bar=20 μm). All data are presented as mean ± SD. *P < 0.05, **P < 0.01, ***P < 0.001, and ****P < 0.0001.


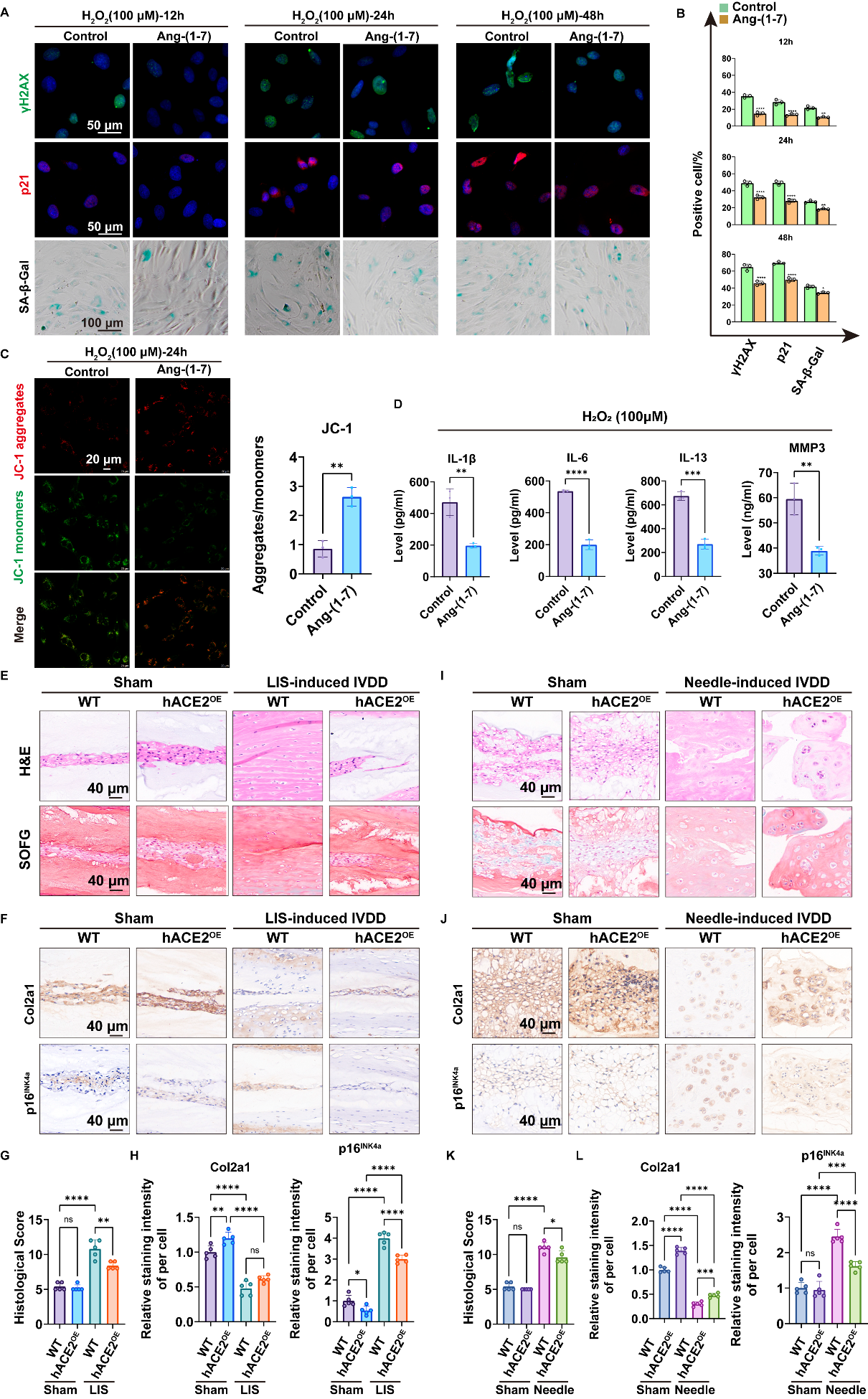


**Figure S7.** **ACE2 overexpression contracted aging and alleviates NPC senescence and IVDD.** (A) Representative images of immunofluorescence of γH2AX, p21, and SA-β-Gal staining in H2O2-treated NPCs with or without Ang-(1-7) (Scale bar=50 μm, 100 μm) (n=3 per groups). (B) The quantification of IF results for γH2AX and expression, and of SA-β-Gal staining was performed. Two-tailed unpaired Student’s t test was used. (C) Representative image and quantitative results of mitochondrial membrane potential in H2O2-treated NPCs with or without Ang-(1-7) (Scale bar=20 μm) (n=3 per groups). (D) ELISA results of the SASP-related cytokines (IL-1β, IL-6, IL-13, and MMP3) content in the supernatant from NPCs in different groups. Two-tailed unpaired Student’s t test was used. (E) Representative images of H&E and SOFG staining of IVD tissue from WT and hAce2OE mice with or without the surgery of lumbar instability (Scale bar=40 μm). (F) Representative images of IHC staining for Col2a1 and p16INK4a of IVD tissue from WT and hAce2OE mice with or without the surgery of lumbar instability (Scale bar=40 μm). (G) Histological score of IVD tissue from WT and hAce2OE mice with or without the surgery of lumbar instability (n=5 per group). (H) The quantification of IHC results for Col2a1 and p16INK4a of IVD tissue from WT and hAce2OE mice with or without the surgery of lumbar instability. (I) Representative images of H&E and SOFG staining of IVD tissue from WT and hAce2OE mice with or without the surgery of tail needling (Scale bar=40 μm). (J) Representative images of IHC staining for Col2a1 and p16INK4a of IVD tissue from WT and hAce2OE mice with or without the surgery of tail needling (Scale bar=40 μm). (K) Histological score of IVD tissue from WT and hAce2OE mice with or without the surgery of tail needling (n=5 per group). (L) The quantification of IHC results for Col2a1 and p16INK4a of IVD tissue from WT and hAce2OE mice with or without the surgery of tail needling (n=5 per group). P values were determined by two-way ANOVA with Tukey's post hoc test. All data are presented as mean ± SD. H&E: Hematoxylin and eosin; SOFG: Safranin-O fast Green; IVD: Intervertebral disc; IHC: Immunohistochemistry; IF: Immunofluorescent staining; NPC; Nucleus pulposus cell. *P < 0.05, **P < 0.01, ***P < 0.001, and ****P < 0.0001.


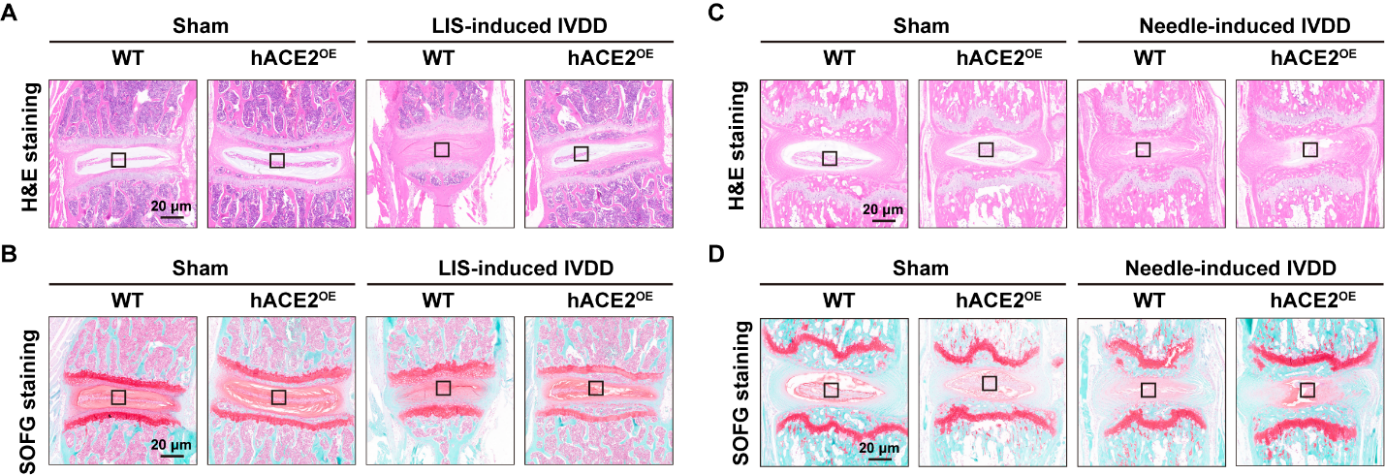


**Figure S8. Histological analysis of IVD from WT and h*Ace2*^OE^ mice with or without the surgery of lumbar instability or tail needling**. (A) Representative images of H&E staining of IVD tissue from WT and hAce2OE mice with or without the surgery of lumbar instability (Scale bar=20 μm). (B) Representative images of SOFG staining of IVD tissue from WT and hAce2OE mice with or without the surgery of lumbar instability (Scale bar=20 μm). (C) Representative images of H&E staining of IVD tissue from WT and hAce2OE mice with or without the surgery of tail needling (Scale bar=20 μm). (D) Representative images of SOFG staining of IVD tissue from WT and hAce2OE mice with or without the surgery of tail needling (Scale bar=20 μm). H&E: Hematoxylin and eosin; SOFG: Safranin-O fast Green; IVD: Intervertebral disc.


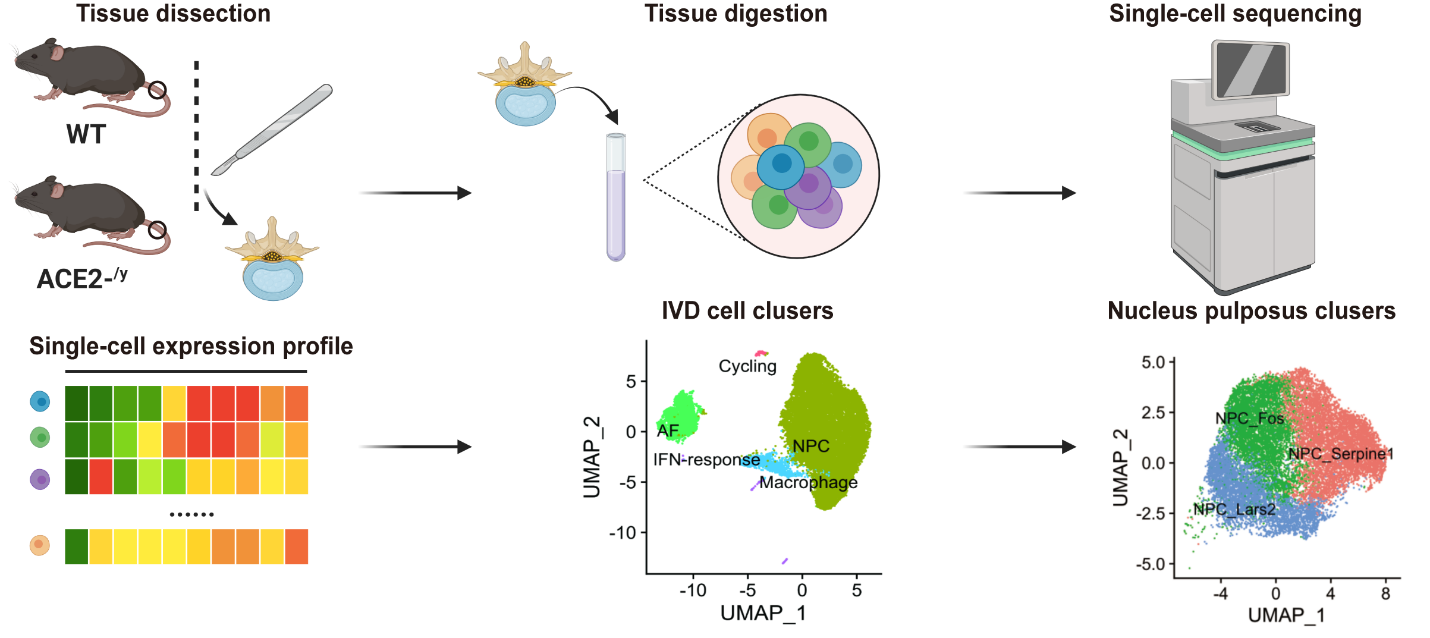


**Figure S9. The illustration of the single cell RNA sequencing (ScRNA-seq) workflow.**


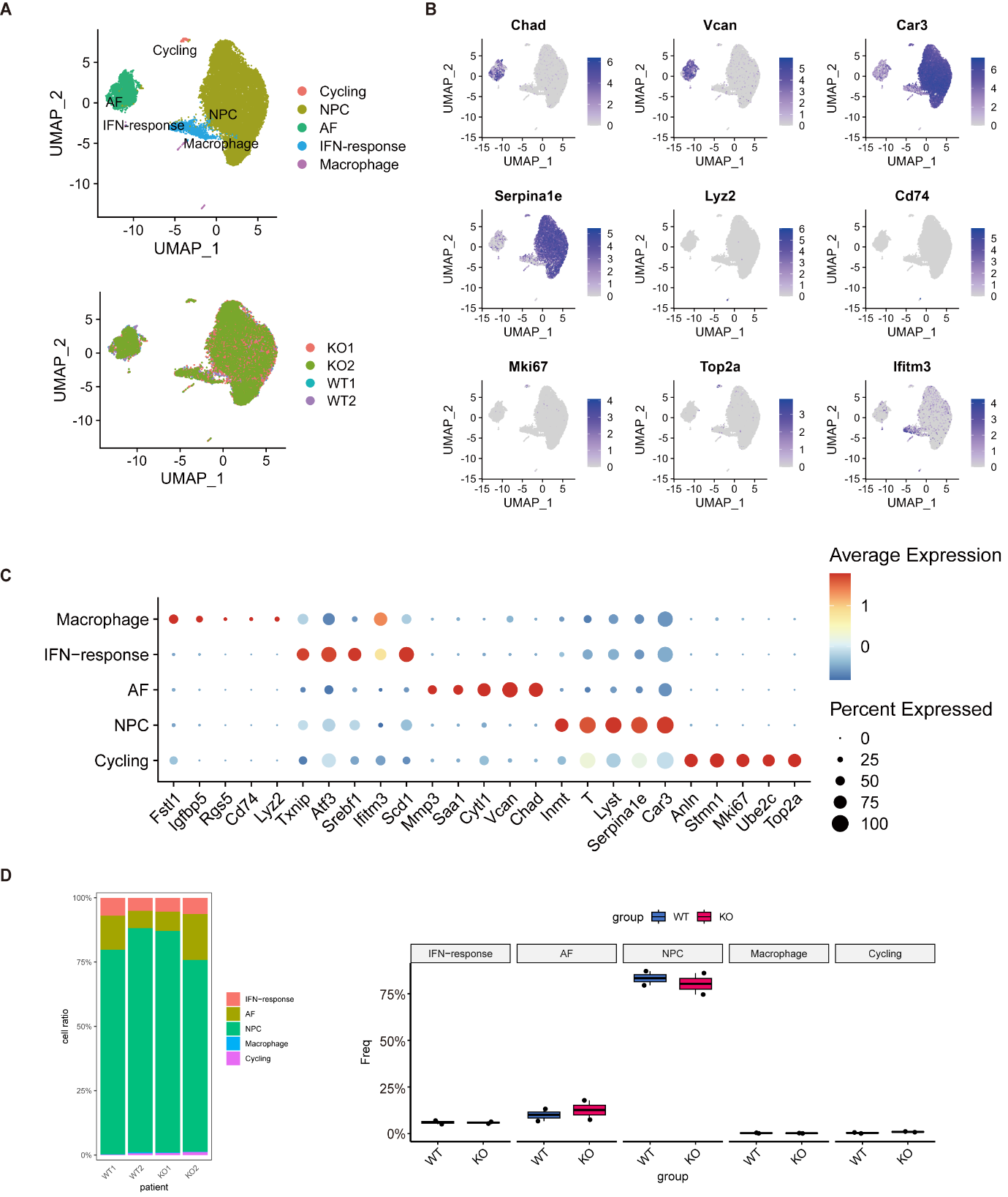


**Figure S10. Single cell RNA sequencing (ScRNA-seq) identified distinct cell subpopulations in IVD from WT and *Ace2*^-/y^ mice**. (A) Visualization of clustering by Uniform Manifold Approximation and Projection (UMAP) plot of cell clusters in IVD samples from WT and *Ace^2^*^-/y^ mice (n=2/group). (B) The differentially expressed genes (DEGs) in each of the cell subclusters. (C) Dot plot of highest DEGs for each major cell type (right axis). Dot color intensity represents the z-score of expression values, and dot size represents percent of cells with at least one UMI detected per gene. (D) Proportion of the five cell subtypes in WT and *Ace2*^-/y^ mice (n=2/group).


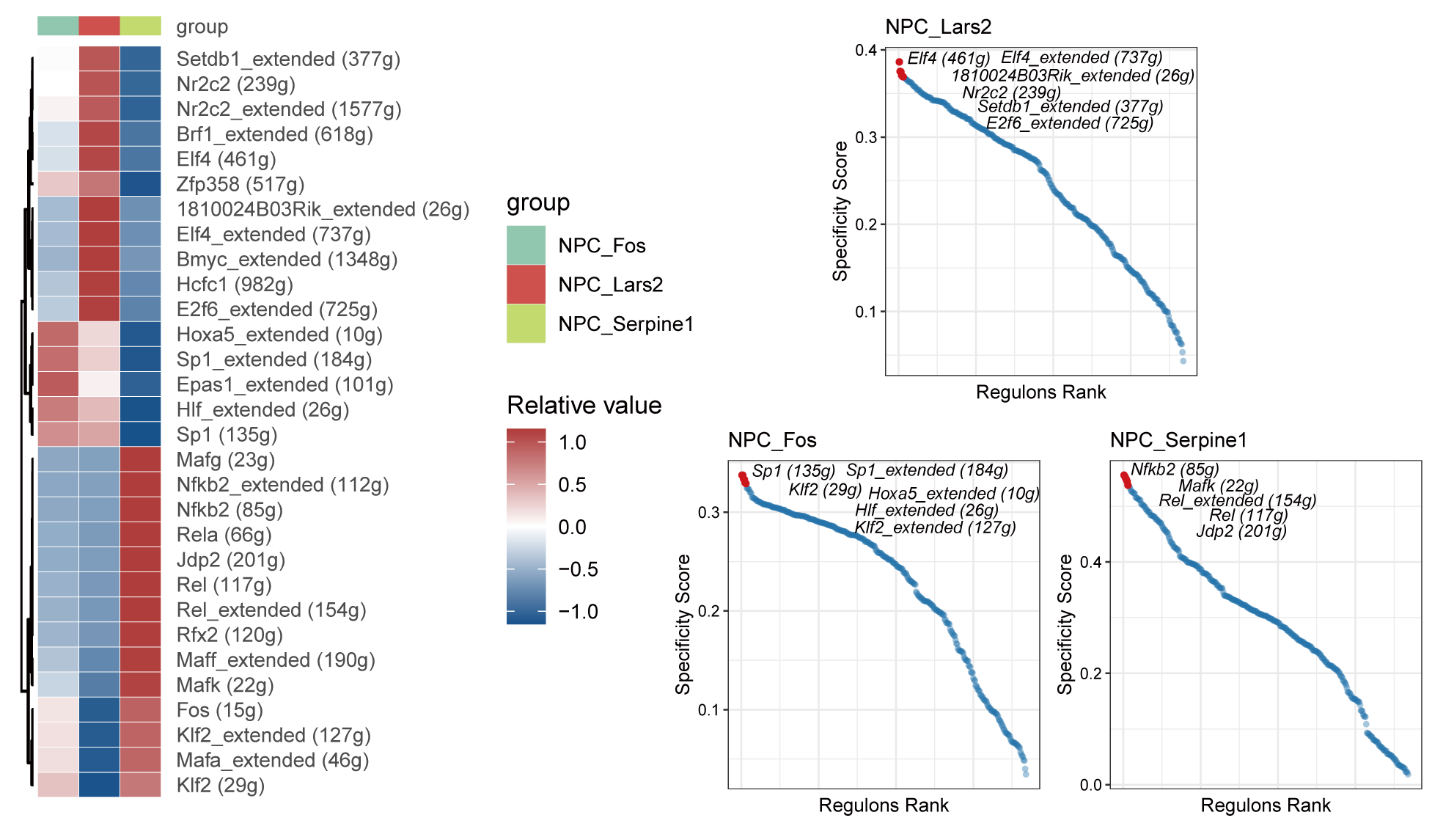
**Figure S11. Analysis of transcript factors in the three NPC subclusters.**


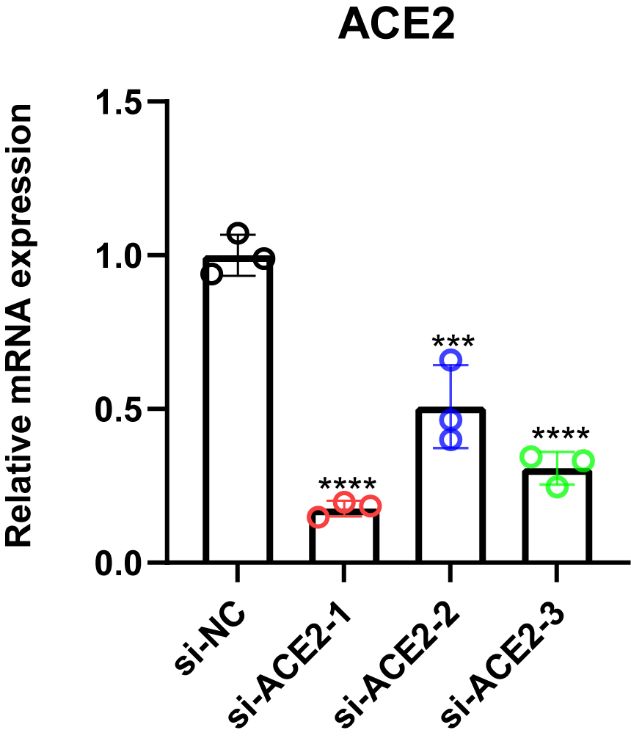


**Figure S12. The selection of siRNA for *ACE2* with the highest knocking down efficiency.** Two-tailed unpaired Student’s t test was used. All data are presented as mean ± SD. ACE2: Angiotensin-converting enzyme 2. *P < 0.05, **P < 0.01, ***P < 0.001, and ****P < 0.0001.


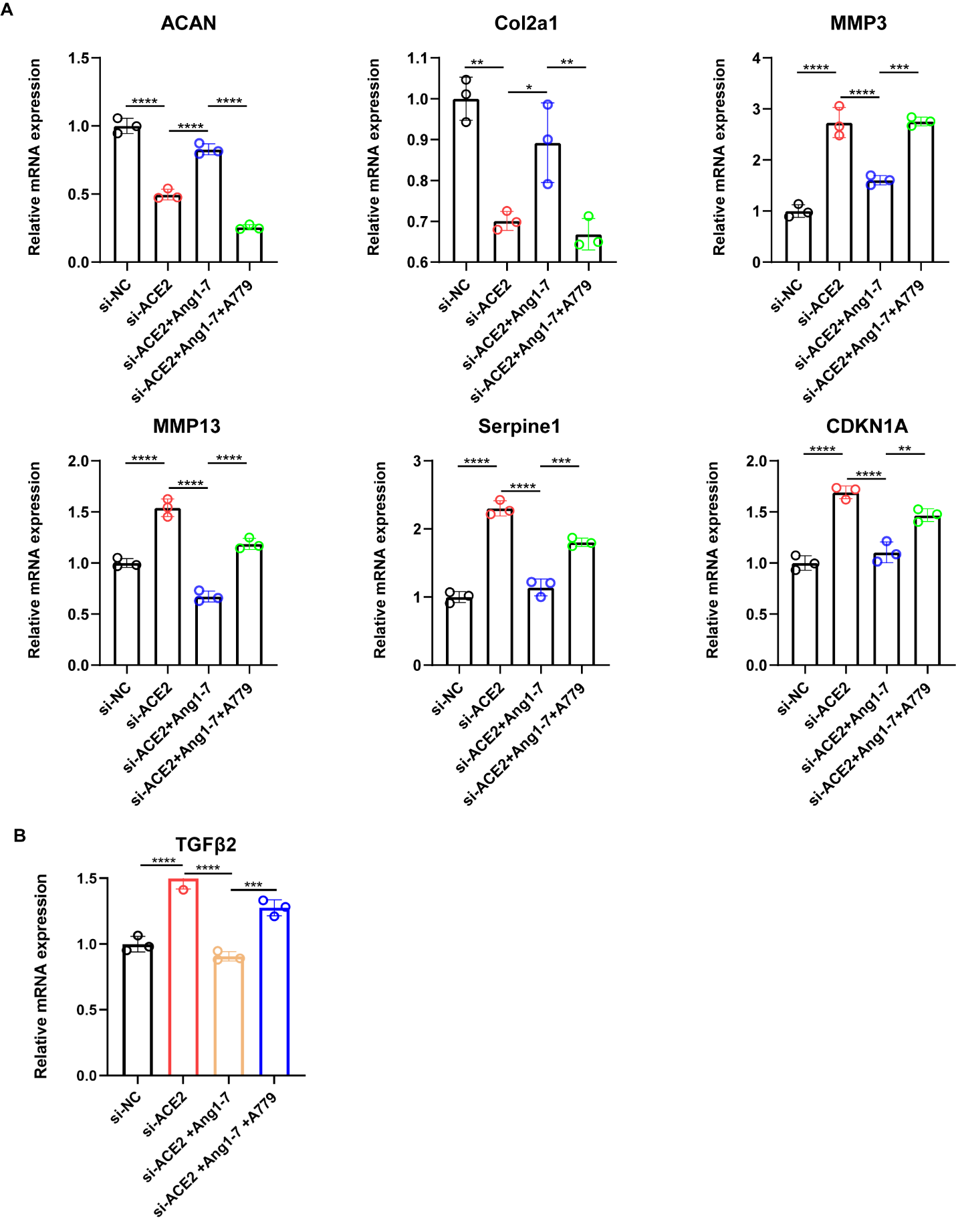


**Figure S13. Ang-(1-7)-MasR axis mediated the protective effects of ACE2 on NPCs against senescence.** (A) The gene expression of *ACAN*, *COL2A1*, *MMP3*, *MMP13*, *IL-1β*, *CDKN1A*, and *CDKN2A* in *ACE2*-knock down NPCs treated by Ang-(1-7) with or without A779, a specific antagonist of MasR (n=3 per group). (B) The gene expression of *TGFβ2* in *ACE2*-knock down NPCs treated by Ang-(1-7) with or without A779, a specific antagonist of MasR (n=3 per group). P values were determined by two-way ANOVA with Tukey's post hoc test. All data are presented as mean ± SD. *P < 0.05, **P < 0.01, ***P < 0.001, and ****P < 0.0001.


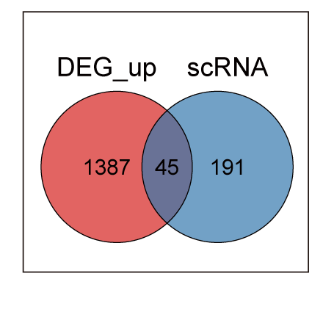


**Figure S14. The Venn diagram of the upregulated differentially expressed genes between RNA-seq analysis in normal and si-ACE2 NPCs and scRNA-seq**.


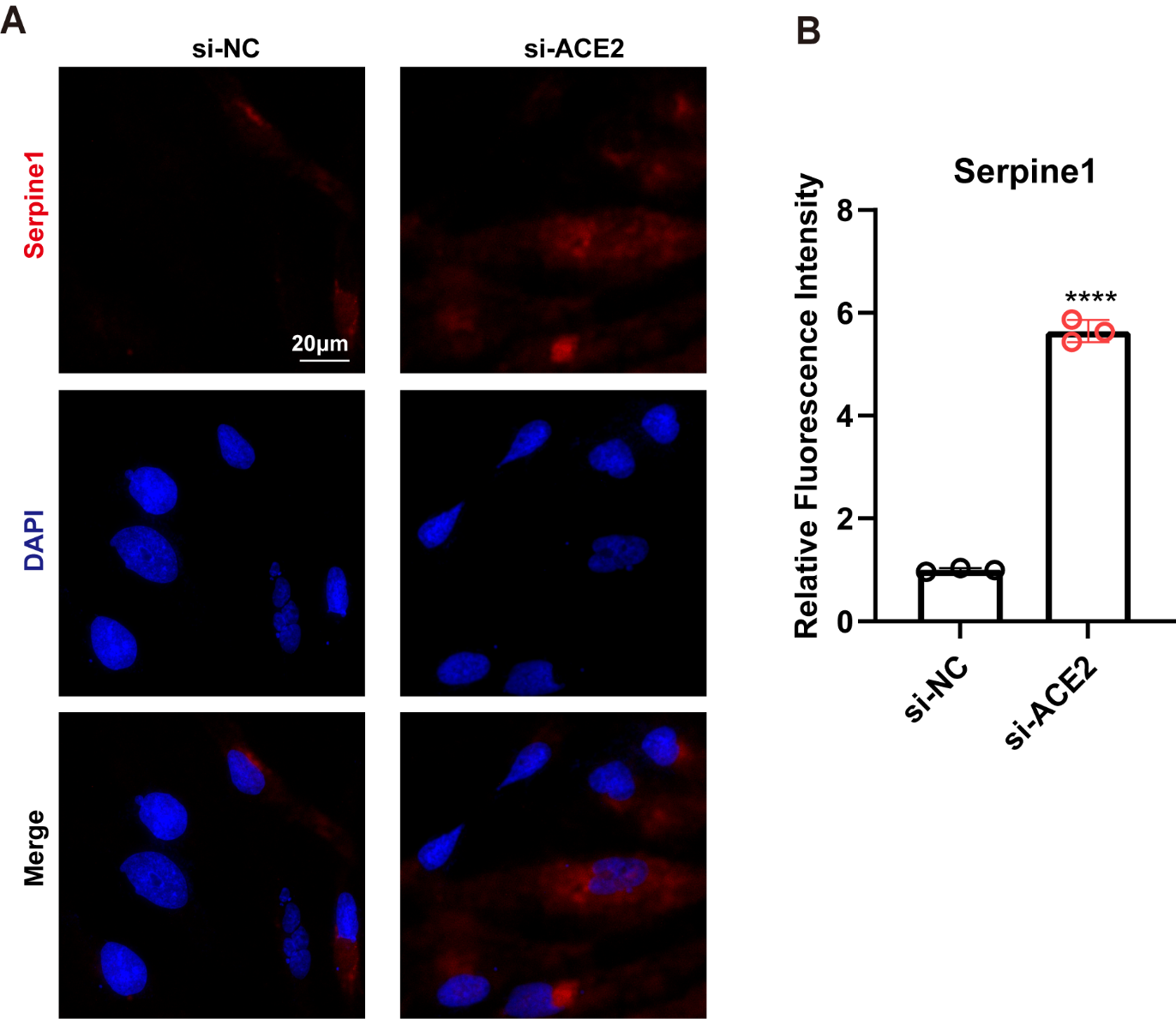


**Figure** **S15. The effects of silencing ACE2 on the expression of SERPINE1.** (A) Representative images of IF staining for SERPINE1 in NPCs with or without ACE2 inhibition (Scale bar=20 μm). (B) Quantitative results of IF staining for SERPINE1 in NPCs with or without ACE2 inhibition (n=3 per group). Two-tailed unpaired Student’s t test was used. All data are presented as mean ± SD. *P < 0.05, **P < 0.01, ***P < 0.001, and ****P < 0.0001.


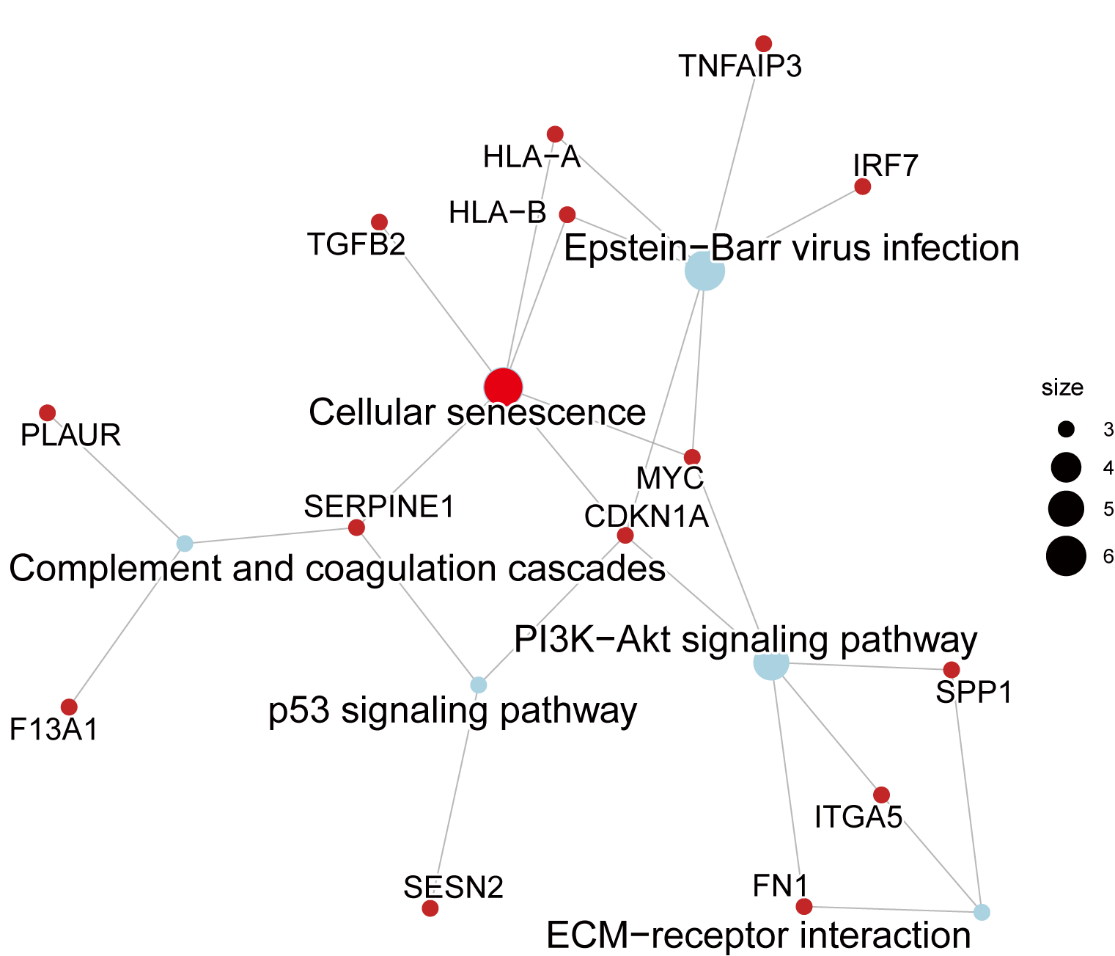


**Figure S16. KEGG analysis also indicated that SERPINE1 related closely to cellular senescence.**


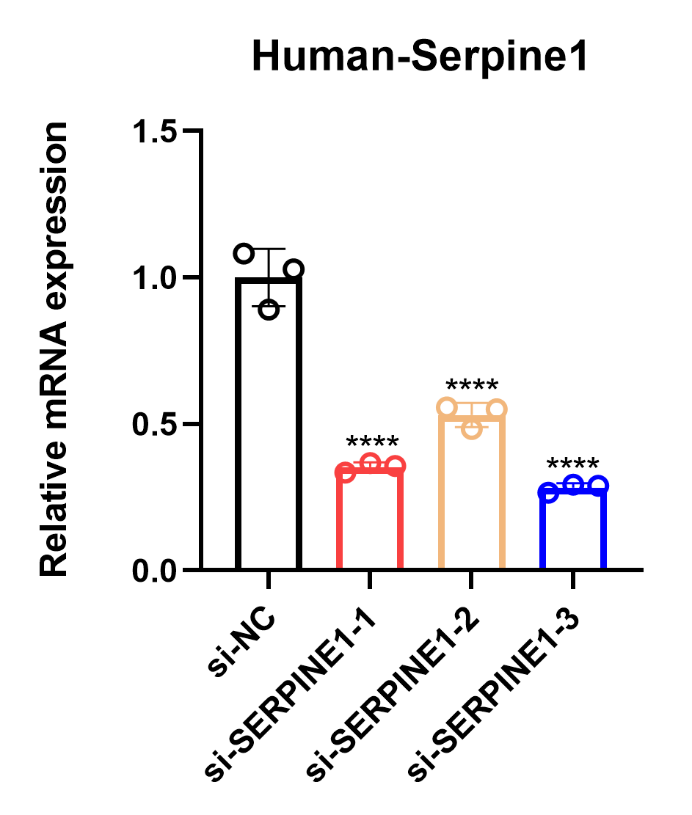


**Figure** **S17. The selection of siRNA for human *SERPINE1* with the highest knocking down efficiency.** Two-tailed unpaired Student’s t test was used. All data are presented as mean ± SD. *P < 0.05, **P < 0.01, ***P < 0.001, and ****P < 0.0001.


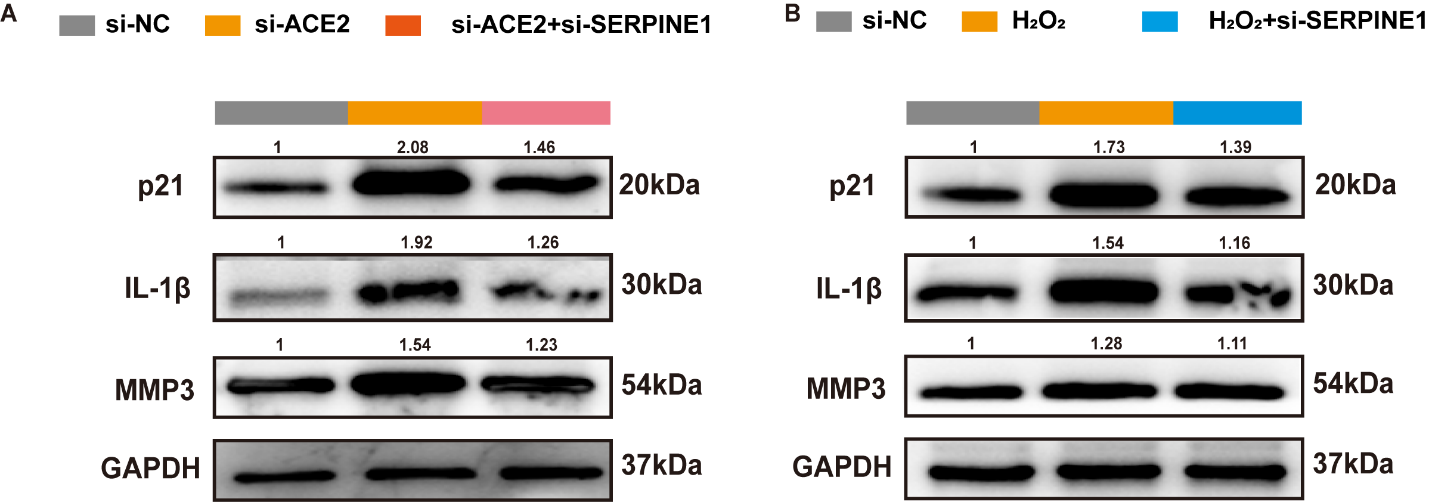


**Figure S18. SERPINE1 mediated senescence and degeneration in ACE2 deficient or H_2_O_2_-treated NPC.** (A) Western blot analysis indicated the protein expression in ACE2-decificent NPCs with or without the inhibition of SERPINE1. (B) Western blot analysis indicated the protein expression in H_2_O_2_-treated NPCs with or without the inhibition of SERPINE1.


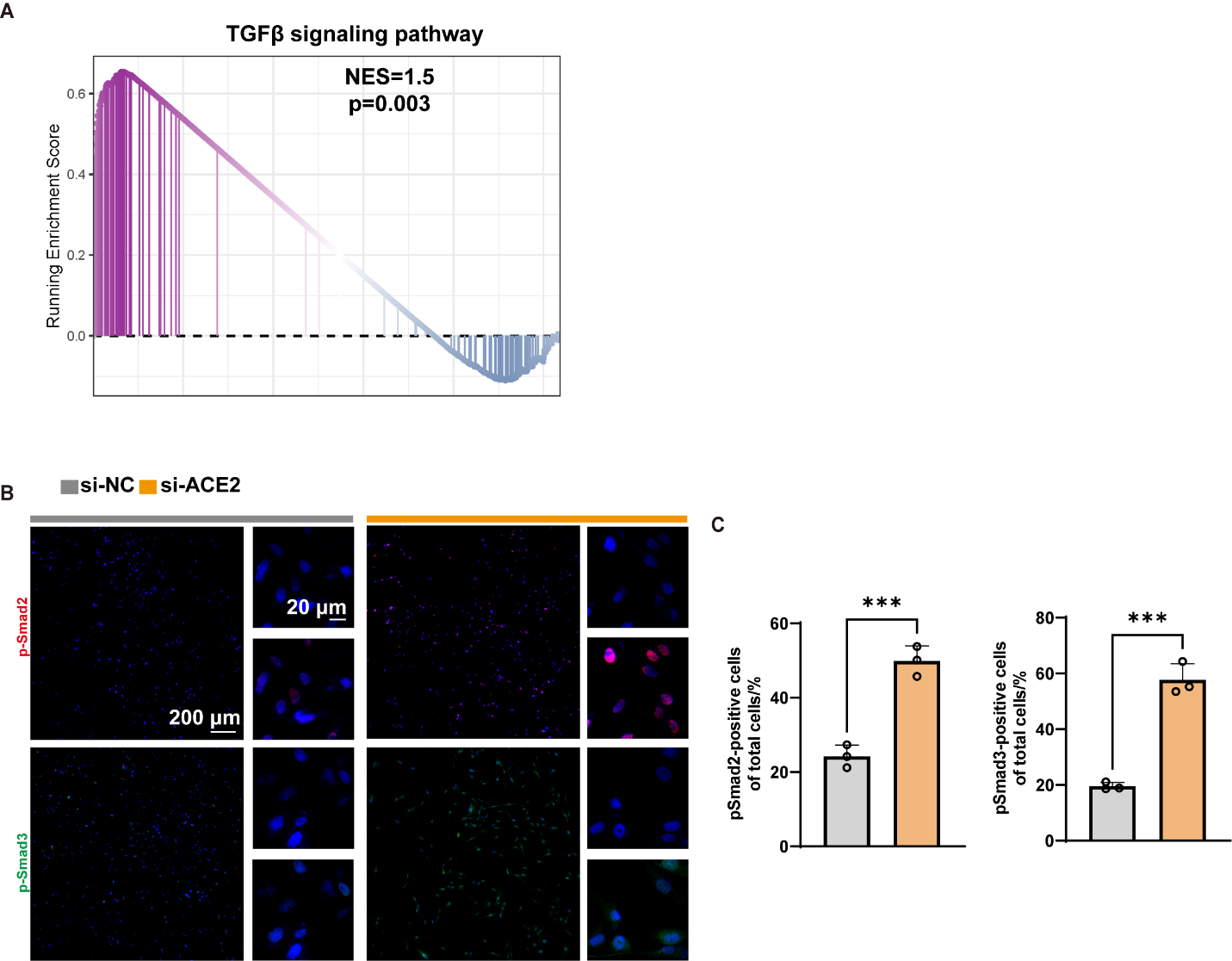


**Figure S19. Inhibiting the expression of *ACE2* activated TGF/Smad2/3 signaling pathway.** (A) GSEA analysis revealed that *ACE2* deficiency contributed to the activation of TGFβ signaling pathway. (B) Representative images of IF staining for p-Smad2/3 in NPCs with or without ACE2 inhibition (Scale bar=200 μm, 20 μm). (B) Quantitative results of IF staining for p-Smad2/3 in NPCs with or without *ACE2* inhibition (n=3 per group). Two-tailed unpaired Student’s t test was used. All data are presented as mean ± SD. *P < 0.05, **P < 0.01, ***P < 0.001, and ****P < 0.0001.


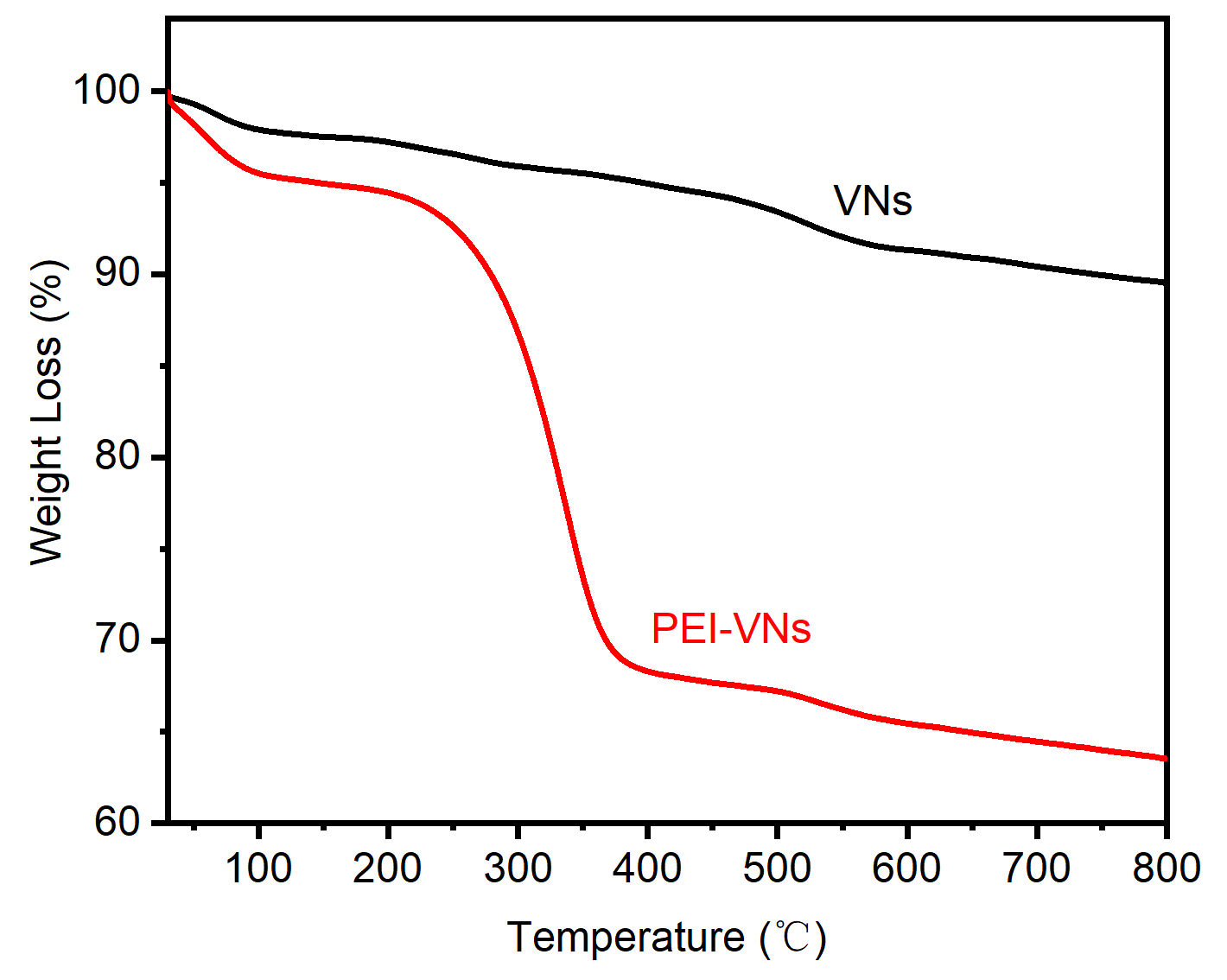


**Figure S20. TGA of VNs and PEI-VNs.**


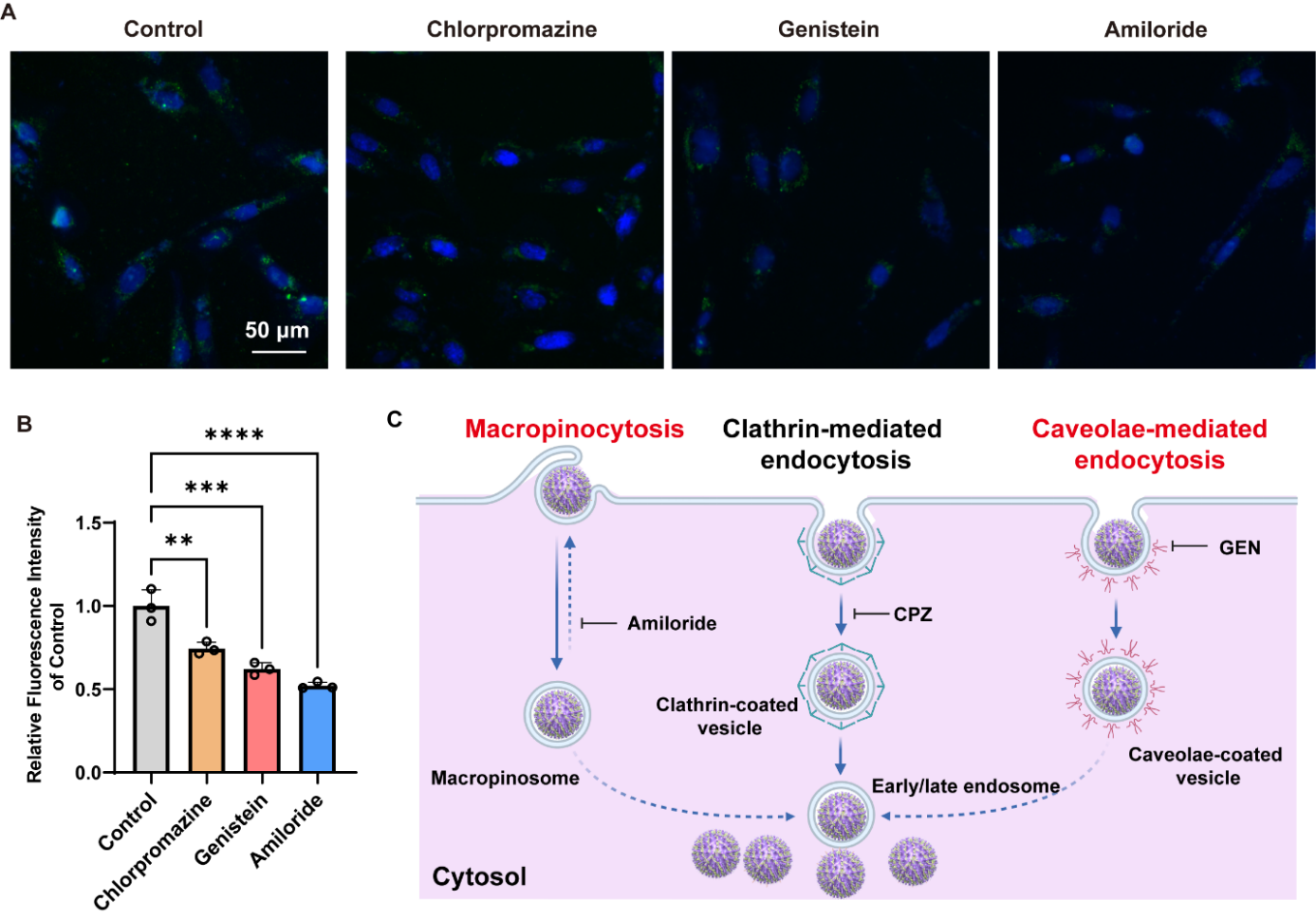


**Figure S21. Probing the mechanisms of cellular internalization of VNs by using inhibitors of specific endocytic pathways.** (A) NPCs were incubated with FITC-labeled VNs for 4h in the absence or presence of inhibitor amiloride, chlorpromazine and genistein, and representative images were shown. (B) Quantitative results of the internalized FITC-labeled VNs in NPCs (n=3 per group). P values were determined by two-way ANOVA with Tukey's post hoc test. All data are presented as mean ± SD. (C) Schematic illustration of intracellular processing of nanoparticles which are regulated by multiple key factors. *P < 0.05, **P < 0.01, ***P < 0.001, and ****P < 0.0001.


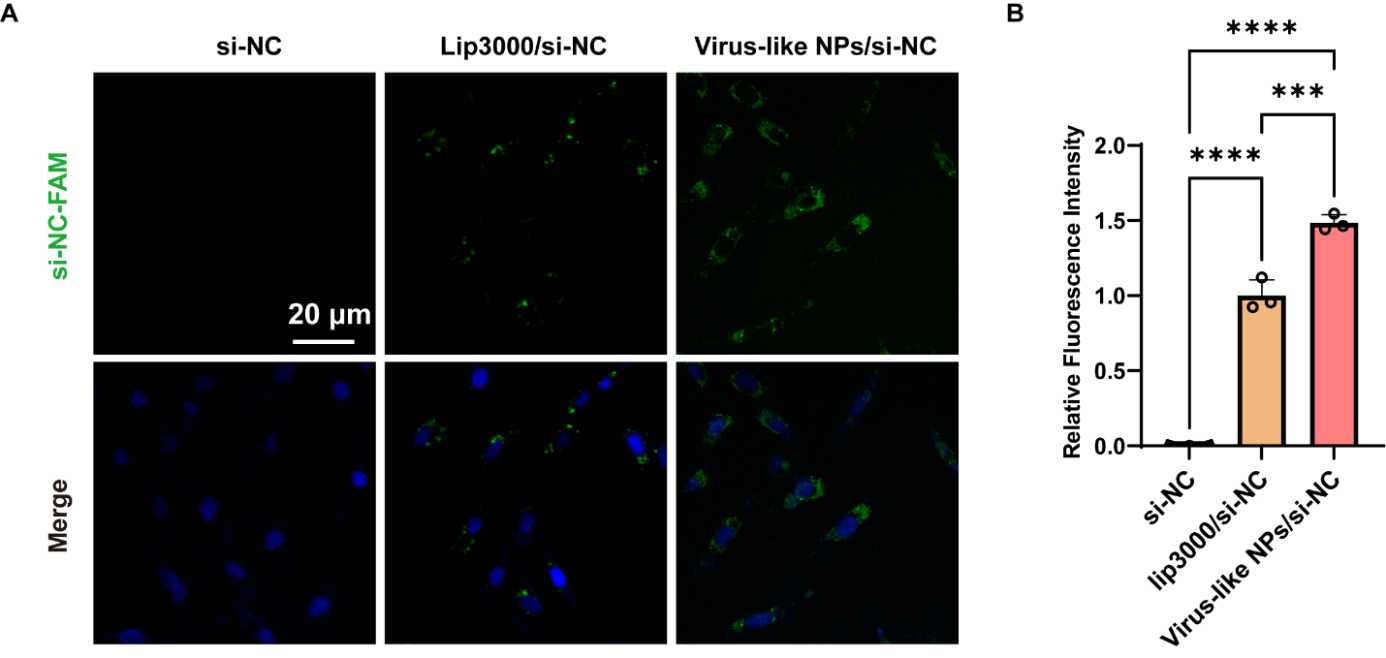


**Figure S22. Assessment of the transfection efficacy of VNs in vitro.** (A and B) Assessment of the transfection efficacy of VNs on NPCs by FITC-labeled si-NC, from which relative fluorescence intensity was analyzed (Scale bar=20 μm) (n=3 per group). P values were determined by two-way ANOVA with Tukey's post hoc test. All data are presented as mean ± SD. *P < 0.05, **P < 0.01, ***P < 0.001, and ****P < 0.0001.


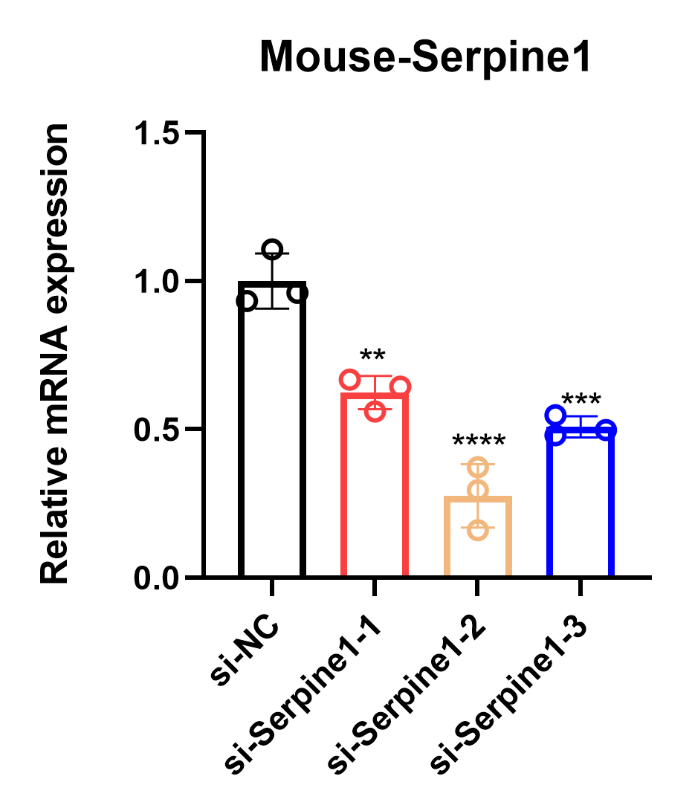


**Figure S23. The selection of siRNA for mouse *Serpine1* with the highest knocking down efficiency.** Two-tailed unpaired Student’s t test was used. All data are presented as mean ± SD. *P < 0.05, **P < 0.01, ***P < 0.001, and ****P < 0.0001.


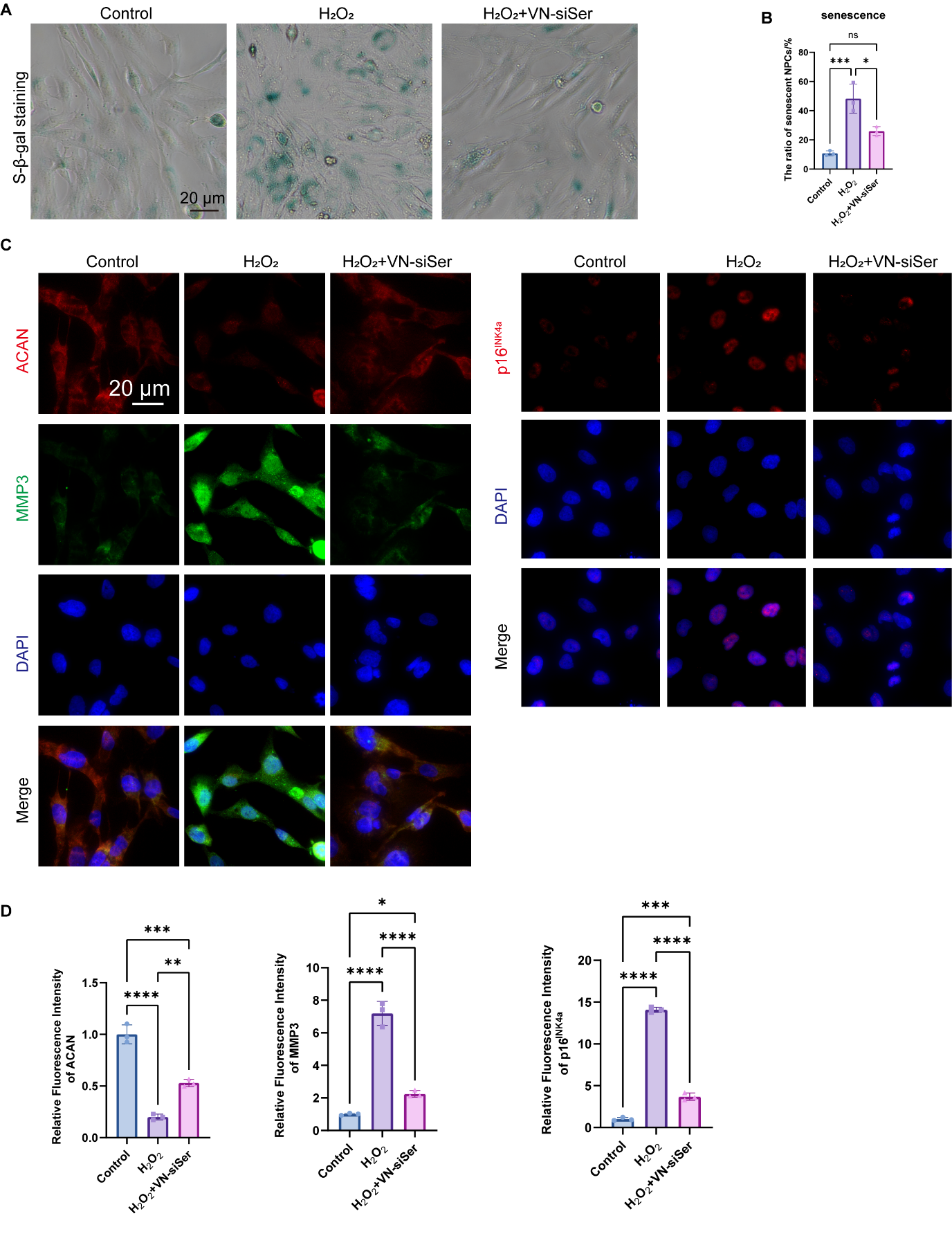


**Figure S24. Inhibiting *Serpine1* using VN-siSer alleviated senescence and SASP in H_2_O_2_-treated NPCs.** (A) S-β-Gal staining of H2O2-treated NPCs with or without the inhibition of Serpine1 via VN-siSer (Scale bar=20 μm). (B) Quantitative results of S-β-Gal staining of H2O2-treated NPCs with or without the inhibition of Serpine1 via VN-siSer (n=3 per group). (C) Representative images of IF staining for ACAN, MMP3, and p16INK4a in H2O2-treated NPCs with or without the inhibition of Serpine1 via VN-siSer (Scale bar=20 μm). (D) Quantitative results of IF staining for ACAN, MMP3, and p16INK4a in H2O2-treated NPCs with or without the inhibition of Serpine1 via VN-siSer (n=3 per group). P values were determined by two-way ANOVA with Tukey's post hoc test. All data are presented as mean ± SD. IF: Immunofluorescent staining; NPC; Nucleus pulposus cell; Serpine1: Serpin family E member 1. *P < 0.05, **P < 0.01, ***P < 0.001, and ****P < 0.0001.


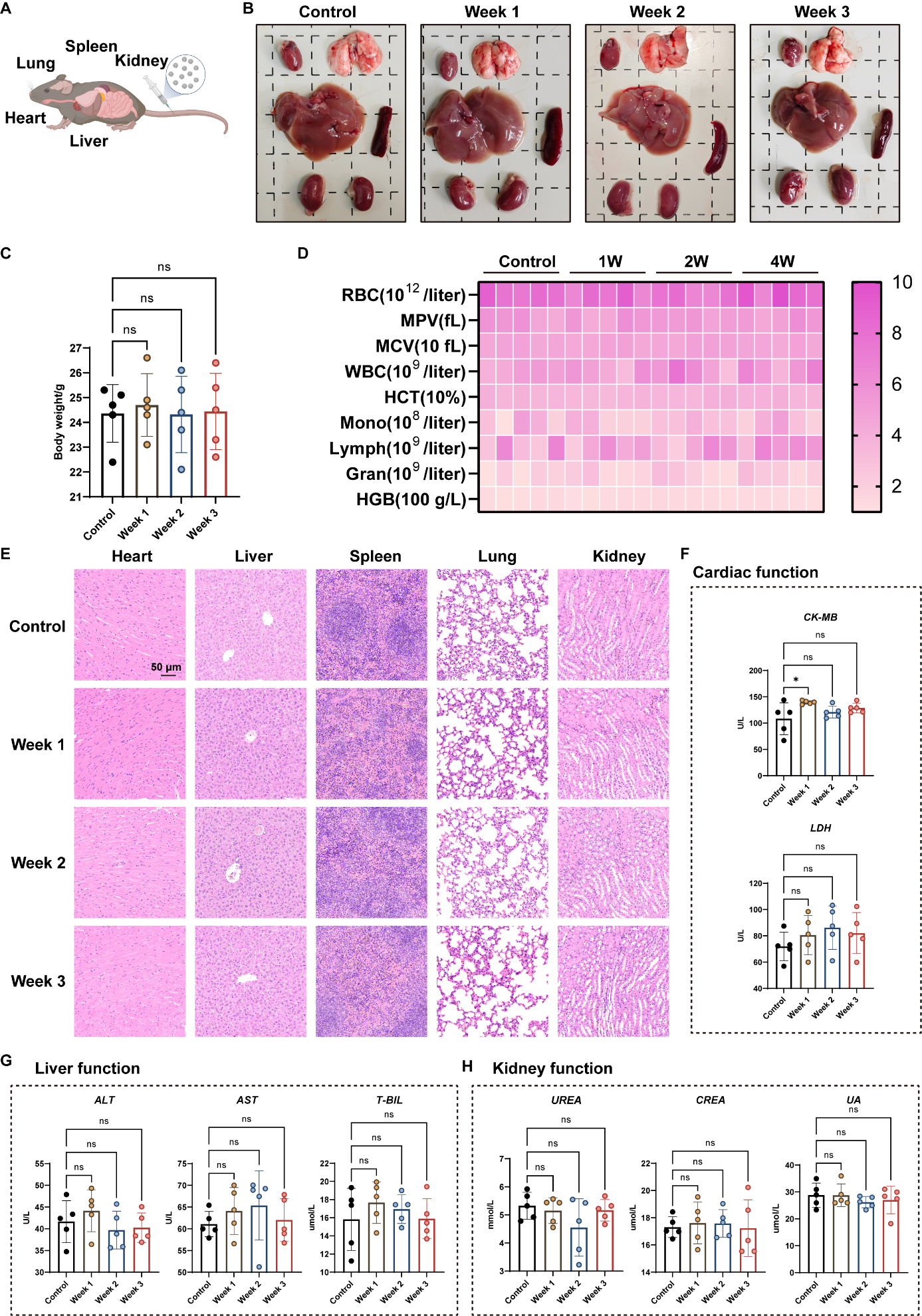


**Figure S25. In vivo biosafety of VNs** (A) Illustration of the collection of the major organs (heart, liver, spleen, lung, and kidney) from mice. (B) The gross morphology of the major organs (heart, liver, spleen, lung, and kidney) from mice at 1, 2, and 3 weeks after the injection of VNs into tail. (C) The body weight of mice at 1, 2, and 3 weeks after the injection of VNs into tail (n=5 per group). Two-tailed unpaired Student’s t test was used. All data are presented as mean ± SD. (D) The results of blood routine examination of mice at 1, 2, and 3 weeks after the injection of VNs into tail (n=5 per group). (E) Representative H&E staining the tissue sections of heart, liver, spleen, lung, and kidney obtained from mice at 1, 2, and 3 weeks after the injection of VNs into tail (Scale bar=50 μm). (F, G and H) Blood biochemical analysis for the function of liver, heart, and kidney of mice at 1, 2, and 3 weeks after the injection of VNs into tail (n=5 per group). Two-tailed unpaired Student’s t test was used. All data are presented as mean ± SD. *P < 0.05, **P < 0.01, ***P < 0.001, and ****P < 0.0001.

**Supplementary Tables**

**Table S1. Basic information of patients involved in this study.**

| No. of patients | sex | Age/years | Diagnosis | Surgical level | Pfirrmann score |
| --- | --- | --- | --- | --- | --- |
| 1 | F | 36 | Lumbar fracture | L1/2 | 2 |
| 2 | M | 54 | Lumbar fracture | L3/4 | 2 |
| 3 | F | 43 | Lumbar fracture | L3/4 | 2 |
| 4 | F | 46 | Tethered cord syndrome | L4/5 | 2 |
| 5 | F | 48 | Lumbar fracture | L2/3 | 2 |
| 6 | F | 32 | Tethered cord syndrome | L2/3 | 2 |
| 7 | F | 72 | Lumbar spinal stenosis | L3/4 | 3 |
| 8 | M | 65 | Lumbar spinal stenosis | L4/5 | 3 |
| 9 | M | 52 | Lumbar spondylolisthesis | L4/5 | 3 |
| 10 | M | 56 | Lumbar spinal stenosis | L5/S1 | 3 |
| 11 | F | 50 | Lumbar spondylolisthesis | L3/4 | 3 |
| 12 | M | 35 | Lumbar spinal stenosis | L1/2 | 3 |
| 13 | F | 68 | Lumbar spondylolisthesis | L1/2 | 3 |
| 14 | F | 60 | Tethered cord syndrome | L4/5 | 3 |
| 15 | M | 37 | Lumbar spinal stenosis | L3/4 | 3 |
| 16 | F | 27 | Tethered cord syndrome | L1/2 | 3 |
| 17 | M | 51 | Lumbar spinal stenosis | L4/5 | 3 |
| 18 | M | 47 | Lumbar spondylolisthesis | L3/4 | 3 |
| 19 | M | 64 | Lumbar spinal stenosis | L3/4 | 3 |
| 20 | F | 64 | Lumbar spondylolisthesis | L4/5 | 3 |
| 21 | F | 29 | Lumbar spinal stenosis | L3/4 | 3 |
| 22 | F | 51 | Lumbar spondylolisthesis | L3/4 | 3 |
| 23 | M | 46 | Tethered cord syndrome | L1/2 | 3 |
| 24 | M | 56 | Lumbar spinal stenosis | L3/4 | 3 |
| 25 | F | 42 | Lumbar spinal stenosis | L4/5 | 3 |
| 26 | M | 49 | Lumbar spinal stenosis | L4/5 | 3 |
| 27 | M | 53 | Lumbar spinal stenosis | L2/3 | 3 |
| 28 | M | 77 | Lumbar spinal stenosis | L3/4 | 3 |
| 29 | M | 51 | Lumbar spondylolisthesis | L2/3 | 3 |
| 30 | M | 75 | Lumbar spondylolisthesis | L4/5 | 3 |
| 31 | M | 55 | Lumbar spondylolisthesis | L2/3 | 3 |
| 32 | M | 45 | Lumbar spondylolisthesis | L1/2 | 3 |
| 33 | F | 52 | Lumbar spinal stenosis | L1/2 | 4 |
| 34 | M | 67 | Lumbar spinal stenosis | L2/3 | 4 |
| 35 | M | 71 | Lumbar spinal stenosis | L3/4 | 4 |
| 36 | M | 45 | Lumbar spinal stenosis | L3/4 | 4 |
| 37 | M | 37 | Tethered cord syndrome | L2/3 | 4 |
| 38 | F | 46 | Lumbar spinal stenosis | L1/2 | 4 |
| 39 | F | 54 | Tethered cord syndrome | L3/4 | 4 |
| 40 | F | 33 | Lumbar spinal stenosis | L2/3 | 4 |
| 41 | M | 42 | Lumbar spinal stenosis | L4/5 | 4 |
| 42 | F | 44 | Tethered cord syndrome | L3/4 | 4 |
| 43 | M | 48 | Lumbar spinal stenosis | L3/4 | 4 |
| 44 | M | 51 | Lumbar spinal stenosis | L3/4 | 4 |
| 45 | F | 55 | Lumbar spinal stenosis | L1/2 | 4 |
| 46 | F | 69 | Lumbar spinal stenosis | L1/2 | 4 |
| 47 | F | 70 | Lumbar spondylolisthesis | L3/4 | 5 |
| 48 | M | 81 | Lumbar spinal stenosis | L1/2 | 5 |
| 49 | M | 65 | Lumbar spinal stenosis | L2/3 | 5 |
| 50 | F | 67 | Lumbar spinal stenosis | L2/3 | 5 |
| 51 | F | 53 | Lumbar spondylolisthesis | L1/2 | 5 |
| 52 | F | 59 | Lumbar spondylolisthesis | L4/5 | 5 |
| 53 | F | 43 | Lumbar spinal stenosis | L1/2 | 5 |
| 54 | F | 57 | Lumbar spinal stenosis | L4/5 | 5 |
| 55 | F | 49 | Lumbar spinal stenosis | L1/2 | 5 |
| 56 | M | 72 | Lumbar spinal stenosis | L3/4 | 5 |
| 57 | M | 37 | Tethered cord syndrome | L3/4 | 5 |
| 58 | F | 61 | Lumbar spinal stenosis | L4/5 | 5 |

**Table S2. Primer sequences for human tissue and cells.**

| Gene name | Gene ID | Reference sequence | Primer Sequence (5'-3') |
| --- | --- | --- | --- |
| *AGGRECAN* | 176 | NC_000015 | F: GGGAAGGCTGCTATGGAGAC  R: ACCTCACCCTCCATCTCCTC |
| *COL2A1* | 1280 | NM_001844 | F: CCAGAAACAACACAATCCGTT  R: ATGGACATCAGGTCAGGTCAG |
| *MMP3* | 4314 | NM_002422 | F: GGTGTGGAGTTCCTGATGTTGGTC  R: AGCCTGGAGAATGTGAGTGGAGTC |
| *MMP13* | 4322 | NM_002427 | F: ATCTGAACTGGGTCTTCCAA  R: GCCTGTATCCCTCAAAGTGAAC |
| *ACE2* | 59272 | NM_001371415.1 | F: CTGCTAAGAAATGGAGCTAATGA  R: GGTGACAGAAGACCAATGGA |
| homo-*SERPINE1* | 5054 | NM_000602 | F: GGAGAGAGCCAGATTCATCAT  R: GAAGTAGAGGGCATTCACCAG |
| mus-*Serpine1* | 18787 | NM_008871 | F: ACTTTACCCCTCCGAGAATC  R: CCTGCTGAAACACTTTTACTC |
| *TGFβ2* | 7042 | NM_001135599 | F: TTCAAGCAGACCGATGTCTAC  R: GCAGCAGGGACAGTGTAAG |
| *CDKN1A* | 1026 | NM_000389 | F: CACTTTGATTAGCAGCGGAAC  R: CAACTACTCCCAGCCCCATAT |
| *IL-1β* | 3553 | NM_000576 | F: GATATGGAGCAACAAGTGGT  R: AGGACAGGTACAGATTCTTTTC |
| *CDKN2A* | 1029 | NM_000077 | F: GATTGAAAGAACCAGAGAGGCT  R: GACCTTCGGTGACTGATGAT |
| *TGFβ2* | 7042 | NM_001135599 | F: AAG AGC AGA AGG CGA ATG  R GCA GCA GGG ACA GTG TAA G |
| *β-Actin* | 60 | NM_001101 | F: AAGGTGACAGCAGTCGGTT  R: TGTGTGGACTTGGGAGAGG |

**Table S3. Histological grading system of intervertebral disc.**

| Category | Score |
| --- | --- |
| Morphology of the NP | Score 0: round shape and the NP constitutes >75% of the disc area;  Score 1: round shape and the NP constitutes 50–75% of the disc area;  Score 2: round shape and the NP constitutes 25–50% of the disc area;  Score 3: round shape and the NP constitutes <25% of the disc area. |
| Cellularity of the NP | Score 0: stellar-shaped cells with a proteoglycan matrix located at the periphery, evenly distributed;  Score 1: partially stellar and partially round cells, more stellar than round;  Score 2: mostly large, round cells, separated by dense areas of proteoglycan matrix;  Score 3: large, round cells, separated by dense areas of proteoglycan matrix. |
| Morphology of the AF | Score 0: well-organized collagen lamellae with no ruptures;  Score 1: inward bulging, ruptured, or serpentine fibers constitute <25% of the af;  Score 2: inward bulging, ruptured, or serpentine fibers constitute 25−50% of the af;  Score 3: inward bulging, ruptured, or serpentine fibers constitute >50% of the af. |
| Cellularity of the AF | Score 0: fibroblasts comprise >90% of the cells;  Score 1: fibroblasts comprise >75–90% of the cells;  Score 2: intermediate;  Score 3: chondrocytes comprise >75% of the cells. |
| Border between the NP and AF | Score 0: normal, without any interruption;  Score 1: minimal interruption;  Score 2: moderate interruption;  Score 3: severe interruption. |
